# Does having a mobile phone matter? Linking phone access among women to health in India: An exploratory analysis of the National Family Health Survey

**DOI:** 10.1371/journal.pone.0236078

**Published:** 2020-07-20

**Authors:** Diwakar Mohan, Jean Juste Harrisson Bashingwa, Nicki Tiffin, Diva Dhar, Nicola Mulder, Asha George, Amnesty E. LeFevre

**Affiliations:** 1 Department of International Health, Johns Hopkins Bloomberg School of Public Health, Baltimore, Maryland, United States of America; 2 Computational Biology Division, University of Cape Town, Cape Town, South Africa; 3 Wellcome Centre for Infectious Disease Research in Africa, Institute for Infectious Disease and Molecular Medicine, University of Cape Town, Cape Town, South Africa; 4 Health Intelligence Initiative, Public Health and Family Medicine, University of Cape Town, Cape Town, South Africa; 5 Division of Epidemiology and Biostatistics, Public Health and Family Medicine, University of Cape Town, Cape Town, South Africa; 6 Bill and Melinda Gates Foundation, New Delhi, Delhi, India; 7 School of Public Health, University of the Western Cape, Cape Town, South Africa; Institute of Economic Growth, INDIA

## Abstract

**Background:**

The disruptive potential of mobile phones in catalyzing development is increasingly being recognized. However, numerous gaps remain in access to phones and their influence on health care utilization. In this cross-sectional study from India, we assess the gaps in women’s access to phones, their influencing factors, and their influence on health care utilization.

**Methods:**

Data drawn from the 2015 National Family Health Survey (NFHS) in India included a national sample of 45,231 women with data on phone access. Survey design weighted estimates of household phone ownership and women’s access among different population sub-groups are presented. Multilevel logistic models explored the association of phone access with a wide range of maternal and child health indicators. Blinder-Oaxaca (BO) decomposition is used to decompose the gaps between women with and without phone access in health care utilization into components explained by background characteristics influencing phone access (endowments) and unexplained components (coefficients), potentially attributable to phone access itself.

**Findings:**

Phone ownership at the household level was 92·8% (95% CI: 92·6–93·0%), with rural ownership at 91·1% (90·8–91·4%) and urban at 97.1% (96·7–97·3%). Women’s access to phones was 47·8% (46·7–48·8%); 41·6% in rural areas (40·5–42·6%) and 62·7% (60·4–64·8%) in urban. Phone access in urban areas was positively associated with skilled birth attendance, postnatal care and use of modern contraceptives and negatively associated with early antenatal care. Phone access was not associated with improvements in utilization indicators in rural settings. Phone access (coefficient components) explained large gaps in the use of modern contraceptives, moderate gaps in postnatal care and early antenatal care, and smaller differences in the use of skilled birth attendance and immunization. For full antenatal car, phone access was associated with reducing gaps in utilization.

**Interpretation:**

Women of reproductive age have significantly lower phone access use than the households they belong to and marginalized women have the least phone access. Existing phone access for rural women did not improve their health care utilization but was associated with greater utilization for urban women. Without addressing these biases, digital health programs may be at risk of worsening existing health inequities.

## Background

Mobile phones are becoming ubiquitous and, increasingly, an important tool in global health programs. [1–3] Mobile phones have the potential to connect clients with heath care providers, provide new avenues of delivering information, optimize data collection, and facilitate health care worker training and communication. [3–7] Despite low-income countries making rapid advances in mobile phone access, a gender gap persists in access to mobile phones among men and women, which may exacerbate inequalities in access to health information, utilization of health services, adoption of health behaviors and in turn, health outcomes. [8,9] Differentials in access to mobile phones among men and women are estimated to range from 2% in Latin America and East Asia, 14% in sub-Saharan Africa, to 26% in South Asia. [10] Further, among women that do own a phone, usage patterns are significantly lower than men’s, particularly in the use of text messages and internet services. [10]

Globally, mobile phone ownership and use among women is known to be influenced by a number of factors at the individual and household level including women’s age, education, socioeconomic status, and geographic location. [10] Cost of handsets and service are reported as the leading barriers to phone ownership among women, while family or spousal permission was a factor for only 3% of women. [10] Additional barriers include low digital literacy, low overall literacy, lack of perceived relevance, safety and security. [10]

Multiple studies support the idea that mobile phones are a tool for economic growth, and empowering women improves the overall wellbeing of families from an economic perspective. [11–13] Empowering more women with mobile phones has the potential to accelerate social and economic development and the same has been extrapolated to health as well. [11,14] Assumptions have been made that mobile phones, by themselves, may increase access to utilization of healthcare, thereby improving health outcomes. [15] More broadly, mobile health (mHealth) interventions have been shown to positively influence gender relations, providing new modes for health communication among couples, and facilitating greater male participation in health areas typically targeted towards women.^14^ However, in some contexts, mHealth interventions may also exacerbate gender inequalities by reinforcing existing power differentials, disempowering women, and placing them at risk of violence. [10,16,17]

Gender inequality and disempowerment of women has a significant impact on reproductive health, maternal health and overall demand for health care, especially in low and middle income settings. [18] In certain conservative settings in India, social norms dictate that women comply with their husband’s or in-laws’ demands. [19–22] These social norm based restrictions have also prevented access to mobile phones for women of reproductive age group for various reasons including concerns of reputational risk, harassment by strangers and mobile phones distracting women from their primary roles as caregivers in the family. [23] Women are also excluded or disadvantaged in relation to decision-making and access to economic and social resources and this, along with restricted mobility due to cultural and social norms, compounds poor health care-seeking patterns. [23–25]

Large gaps exist between men’s and women’s ownership of mobile phones and these gaps vary widely among different states of India. [23] Factors like education and wealth appear to be highly influential on the magnitude of these gaps, not to mention the interaction with urban-rural residence. [23] In spite of the potential mobile phones and digital health programs may have in improving women’s health, key questions about factors influencing access for women of reproductive age and influence, if any, of mobile phone access on healthcare behavior and health outcomes remain unanswered.

**Fig 1:**
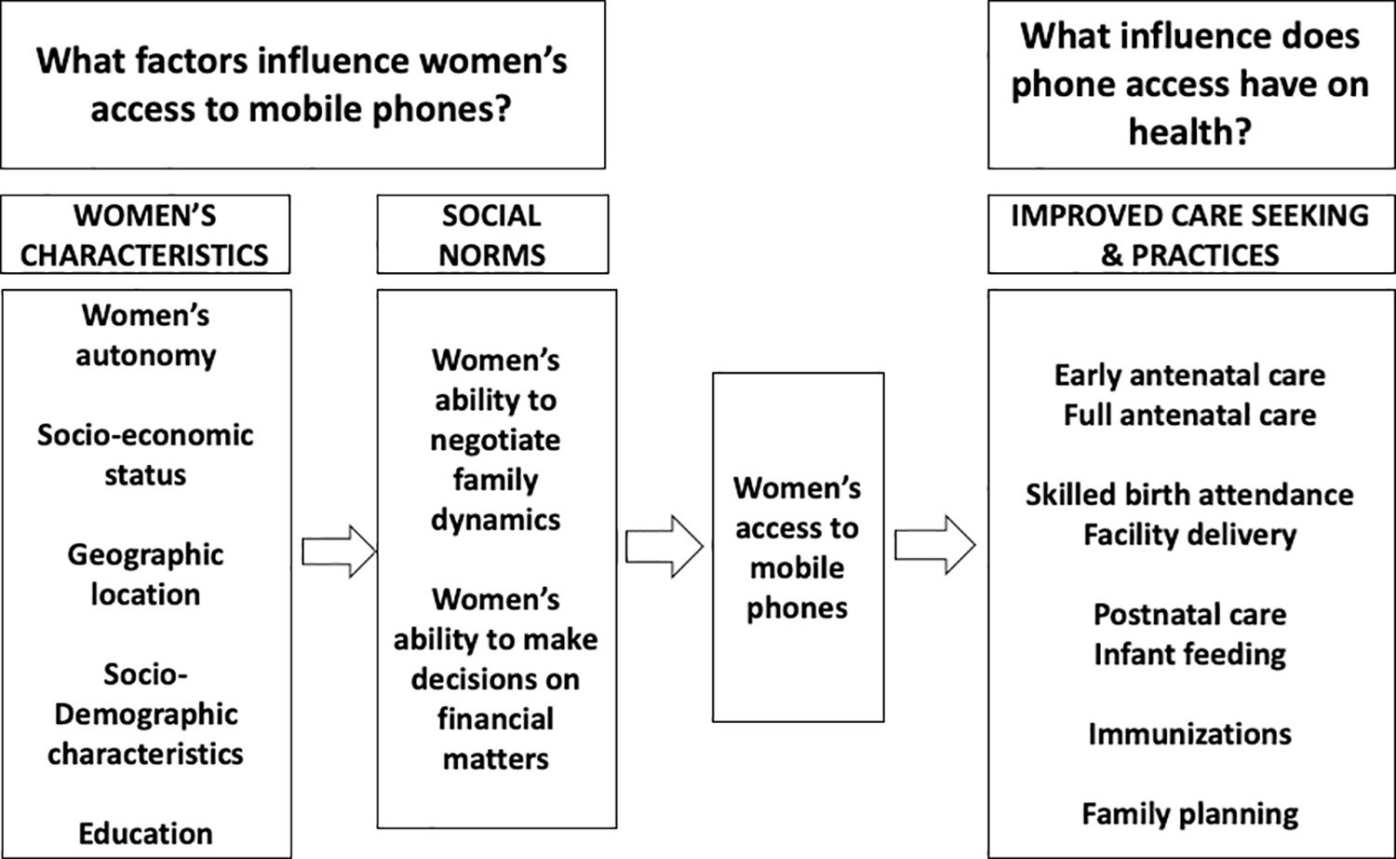
Conceptual framework for understanding factors underpinning women’s access to mobile phones and linkages between phone access and health behaviors.

In this study, we used the recent National Family Health Survey 2015 (NFHS4) from India to explore the intersection between gender, mobile phone access and health behaviors. Guided by the framework in Fig 1, we had the following objectives: 1. To assess the gap in mobile phone access and its associated factors between households and women of reproductive age group in India; 2. To examine the association of phone access among women of reproductive age on health behaviors; 3. To decompose the gaps in prevalence of health behaviors into components explained by gap in background characteristics and those attributable to gap in phone access.

## Methods

### Data and sample

**Fig 2:**
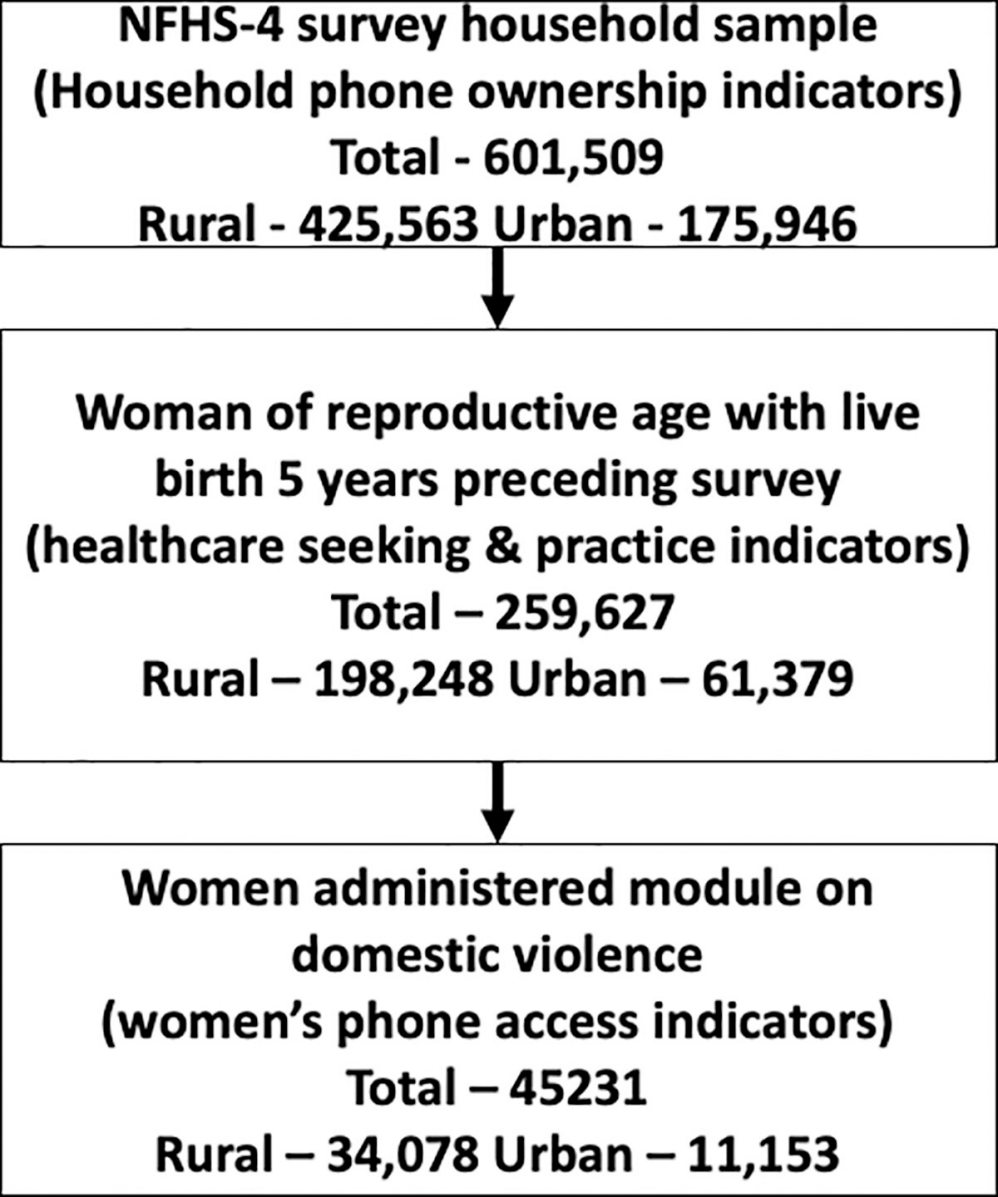
Flowchart for study sample from NFHS 2015–2016.

Data used in this analysis were drawn from the most recent (fourth round) of the Demographic and Health Survey (DHS) for India, also known as the National Family Health Survey-4 (NFHS-4) conducted in 2015–2016. NFHS-4 was carried out by ICF International under the stewardship of the Ministry of Health and Family Welfare (MoHFW), Government of India. The full survey sample includes 699,686 women from 601,509 households (425,563 from rural areas and 175,946 households from urban) with a response rate of 98%. The survey is designed as a two-stage sample design: In stage 1, the Primary Sampling Units (PSU) are villages in rural areas (selected with probability proportional to size); and Census Enumeration Blocks (CEB) in urban areas; in second stage, a random sample of 22 households in each PSU or CEB is selected, respectively. For analysis, an NFHS-4 cluster refers to either a PSU or a segment of a PSU selected at stage 1 of the survey. The data is hierarchical in nature with PSUs nested within districts, and districts nested within states. More details are available elsewhere. For the analysis presented, we used the household mobile phone ownership data from 259627 households (198,248 from rural areas and 61,379 households from urban), where a woman of reproductive age had recently experienced a pregnancy that resulted in a live birth, in the five years preceding the survey. The primary indicator of interest for this study was the access to a mobile phone as reported by these women. The access to a mobile phone is defined as a “yes” to the question “Do you have any mobile phone that you yourself use?”. The female mobile phone access data are only available through an additional module on domestic violence that is administered to a sub-sample of 45,231 women (34,078 from rural areas and 11,153 households from urban). The analysis of the gap in health indicators between women with and without phone access is restricted to this sample. Fig 2 presents a flowchart of the sample used in the analysis.

### Variables

To assess the gap in the reported availability of mobile phones for women of reproductive age, we used the household’s reported ownership of a mobile phone from the asset module in the household questionnaire and compared it with the woman’s reported phone access from the domestic violence module of the women’s questionnaire. The outcomes of interest are indicators of utilization of health care, which are based on the self-reported responses of women surveyed as part of the NFHS survey described above (Table 1). These indicators were chosen because of their importance in the continuum of care for Maternal Neonatal & Child Health (MNCH) as seen in their use by Countdown 2030. The explanatory variables of interest include group level characteristics like the state of residence, classification of the state as an Empowered Action Group (EAG) state, and Urban/ Rural strata. Individual characteristics under consideration are age in years (15–24, 25–34,35–49), religion (Hindu, Christian, Muslim, Other), caste (General category / No caste, Other Backward castes, Schedule castes, Schedule tribes), parity of the woman (Two children, More than 2, One child), educational attainment (No education, Primary, Secondary, Higher), and wealth status (quintiles of wealth score). Multimedia exposure was classified as some exposure if the woman reported watching TV or listening to the radio or reading a newspaper at least once a week. The classification of an EAG state is based on the Government of India's classification of states based on their need for special attention. Women were classified as having participated or not participated in making health decisions for themselves. Women were classified into a dichotomous variable if they said that husbands were justified in beating their wives for specified reasons or not justified for any reason. The women’s wealth status is based on the household’s wealth score derived from a Principal Components Analysis (PCA) of the household assets. [26,27] This variable is part of the recoded dataset provided by NFHS and more details on their calculations can be found elsewhere. [26,27] Household mobile phone ownership is available from the module on the household. The data on phone access for a woman, reported ability of the woman, decision making in the household and partner communication to read an SMS are available from the sub-sample interviewed with the domestic violence module of the women’s questionnaire.

**Table 1:** Health outcomes indicators used in the multilevel models.

Outcome	Description
Early ANC	First ANC visit during the first trimester of the pregnancy (< = 12 weeks)
Full ANC	At least four ANC visits AND at least one tetanus toxoid (TT) injection AND iron folic acid tablets or syrup taken for 100 or more days
Facility delivery	Delivery occurring at a health facility–public or private
Skilled birth attendance	Births assisted by a doctor, nurse, LHV, ANM, or other health personnel
Postnatal Care	At least one contact with a health provider during the 24 hours after delivery
Modern contraceptive use	Reported use of at least one modern contraceptive method (includes condom–male& female condoms, pills, injectables, implants, intrauterine devices, male & female sterilization, female diaphragms (including spermicides), Lactational Amenorrhea Method
Unmet need for family planning	Women currently married or in union who are fecund and who desire to either terminate or postpone childbearing, but who are not currently using a modern contraceptive method. Unmet need for spacing + Unmet need for limiting
Full immunization	Children are fully immunized if they have received BCG, measles, and 3 doses each of polio and DPT
Children under age 6 months exclusively breastfed	Exclusive breastfeeding means that the infant receives only breast milk. No other liquids or solids are given–not even water–with the exception of oral rehydration solution, or drops/syrups of vitamins, minerals or medicines.

### Analysis

All data analyses were performed using R version 3.4.1. [28]

#### Estimates of phone access

The estimates of household ownership, and woman’s phone access are presented as percentages with 95% confidence intervals (CI) across different levels of the explanatory variables. These estimates are adjusted for survey design with appropriate weights provided as part of the NFHS dataset using a robust variance estimator for the confidence intervals. The estimates are derived using the *survey* package version 3.34 in R. [29] The dot plots are based on the prevalence of the household mobile phone ownership and women’s access to phone indicators, adjusted for the survey design with weights. The plots are constructed using the *ggplot2* package in R. Due to sample size considerations, we have combined the union territories into one group. Since the survey was carried out in 2015, Jammu & Kashmir have been presented as a single unit due to the design of the survey.

#### Multilevel models

Multilevel logistic regression models were applied to explore the association of various health outcomes of interest (Table 1) with phone access as reported by women. The models were adjusted for various predictors like: 1) Background demographic, socio-economic and cultural characteristics including age, educational attainment, household socio-economic status, religious affiliation and parity; 2) Media exposure–Defined by the frequency of reading a newspaper or listening to radio or watching TV; 3) EAG classification of states.

The multilevel model is necessary since the data suggests considerable state and district level variance in the different health outcomes. The data have a hierarchical structure with women nested within clusters, which are in turn nested within districts and states. In the multilevel analysis, states are the highest (fourth) level, while districts within states constitute the third level. The general form of the four-level logistic regression model used may be expressed as

$$\log\left(P_{ijks}\right)=X_{ijks}*\beta +u_{jks}+v_{ks}+w_{s}$$

where *P*_*ijks*_ is the probability of an outcome for an individual *i*, in the *j*th cluster in the *k*th district in the sth state; X_ijks_ is the vector of covariates which may be defined at the individual, district or state level; *β* is the associated vector of regression parameter estimates; and the quantities, *u*_*jks*,_ v_ks_ and w_s_ are the residuals at the cluster, district and state levels with normal distribution of mean zero and variances $\sigma_{u}^{2},\;\sigma_{v}^{2}$ and $\sigma_{w}^{2}$ respectively. The multilevel models were analyzed using the *lme4* package in R.

#### Decomposing differences in health

While the multilevel models analyzed evidence for the correlational effects of phone access on different health outcomes, it cannot explain how much of the gap was explained by each of the baseline characteristics. The Blinder-Oaxaca (BO) decomposition was used to decompose any differences in prevalence of health care utilization between women with and without phone access–i.e, understand the magnitude of the gap attributable to the various baseline characteristics. The details of the method and its use in equity analysis have been addressed elsewhere [30]. The prevalence gap between the two groups can be decomposed into 2 main components: (1) the percentage attributable to different levels of the explanatory factors between women with and without phone access (known as the endowment, or explained effect) and (2) the percentage attributable to explanatory factors having differential effects on health outcomes in the two groups (the unexplained /coefficient effect). If outcome *y* is regressed on a set of *k* determinants *x*, *p representing phone access and q lack of phone access*, the gap between the mean values of outcomes for the phone access group, *y*^*p*^, and the group without phone access *y*^*q*^, can be calculated as:

$$y^{p}–y^{q}=\Delta x\beta^{q}+\Delta \beta x^{q}+\Delta x\Delta \beta$$

where *x*^*p*^ and *x*^*q*^ are the average explanatory variables for the groups with and without phone access, respectively; *β*^*p*^ and *β*^*q*^ denote the coefficients of explanatory variables for the above mentioned two groups, respectively; and *Δx* = *x*^*p*^-*x*^*q*^ and *Δβ* = *β*^*p*^-*β*^*q*^. The mean difference in the outcome variable was divided into 3 components: (1) the percentage attributable to different levels of the explanatory factors between the groups with and without phone access (explained components or endowments, *Δxβ*^*q*^), (2) the percentage attributable to explanatory factors having differential effects on poor outcomes between the two groups (the response or coefficient effect, *Δβx*^*q*^), and (3) the percentage attributable to the interaction between the difference in the mean value of ‘endowments’ and their coefficients (*ΔxΔβ*). We modeled the outcome variables as probabilities of the health indicators. The decomposition analysis was performed using the *General Oaxaca* package in R. The three fold Blinder-Oaxaca decomposition with the extension for binomial distribution proposed by Bauer and Sinning was performed with bootstrapped standard errors calculated for estimates of the confidence intervals [31]. Since the predictors are specified as categorical variable dummies, estimates are adjusted to be invariant with respect to the omitted baseline category. In our analysis, the baseline group is assumed to be urban women of age 15–24, Hindu from the poorest quintile and general category of caste with no education and 1–2 children. The decomposition results are presented visually as a bar chart, with the total gap in health utilization indicators between women with and without phone access split into the percentage of endowment, coefficient and interaction components.

#### Ethical approval

The study is based on the NFHS data for India which is an anonymous publicly available dataset with no identifiable information on the survey participants. For the original survey, respondents provided informed consent. Ethical approval for analyses was obtained from the University of Cape Town’s Human Research Ethics Committee.

## Results

### Household ownership and women’s access of mobile phones

**Fig 3:**
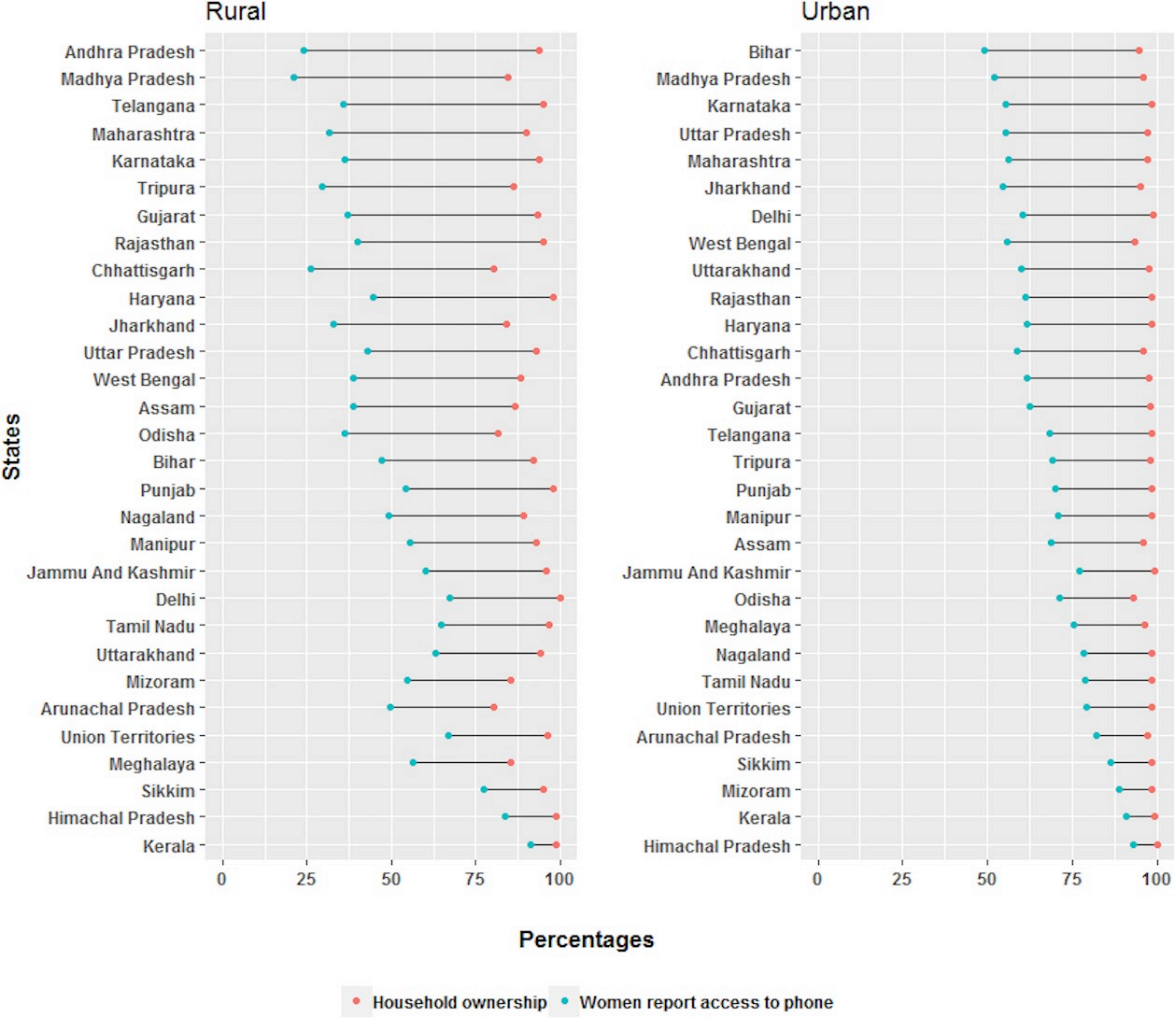
Differentials in rural and urban household ownership of mobile phones and women’s reported access by state. States are listed by the descending order of the gap between household ownership and women’s access.

Overall for India, mobile ownership at the household level was 92·8% (95%CI: 92·6–93·0%): 91·1% (90·8–91·4) rural and 97·1% (96·7–97·3) urban. Women’s access to mobile phones was 47·8% (46·7–48·8) overall, with women’s access reported to be 41·6% (40·5–42·6) in rural areas and 62·7% (60·4–64·8) in urban. State level variations in the mobile access gap for women were observed in both urban and rural settings, with the greatest gaps occurring in the rural areas for all states (Fig 3). Across States, state of Andhra Pradesh had the largest rural gap (69.7%) while the urban gap was greatest in Bihar (45.3%). Kerala and Himachal had the smallest gaps both for rural (7.6%, 15.1%), and urban (8.2%, 7%) areas.

**Fig 4:**
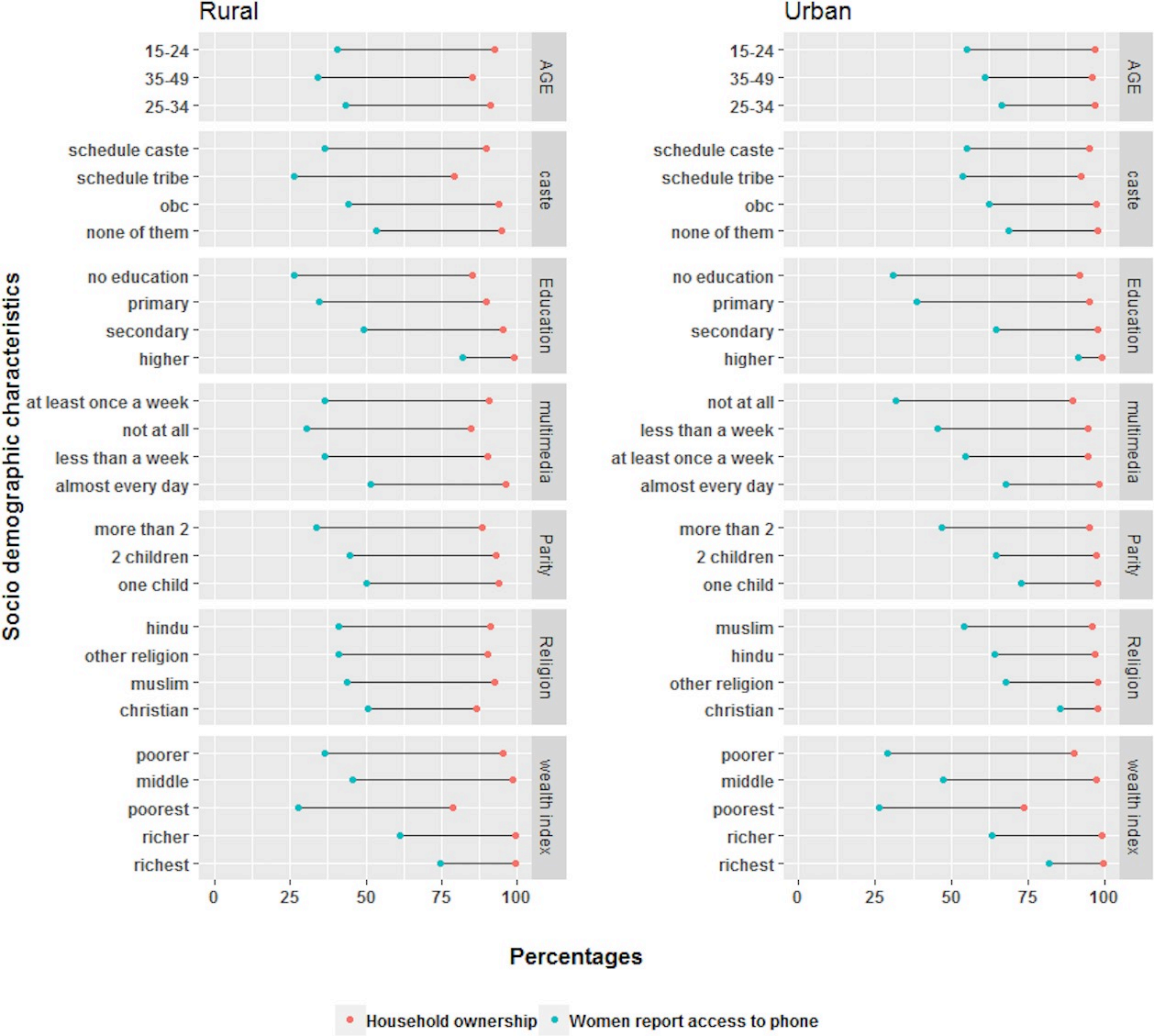
Differentials in rural and urban household ownership of mobile phones and women’s reported access by socio-demographic characteristics. Categories are listed by the descending order of the gap between household ownership and women’s access.

Across socio-demographic characteristics, Christian women report the greatest access compared to all other religions in both urban (85.7%) and rural (50.6%) settings (Fig 4). Women from the poorest quintile have a third of the access of the richest and the gap is similar across rural (27.8% vs 74.8%) and urban (26.3% vs 82.0%) settings. Scheduled castes and tribes have the least access among the caste groups with the gap being considerably smaller in the urban areas than rural areas. Women’s age showed a J curve with bell curve with the youngest and oldest age groups having the least access to mobile phones across rural and urban with a peak around the 30–34 age. Woman’s education shows a large gradient with the difference in access between women with no education and higher education of 55.3% and 61.0% in rural and urban settings, respectively. Detailed estimates of household ownership of phones, women’s access to phones and their reported ability to read text messages (SMS) are provided in S1 Appendix.

Table 2 explores the association between decision making and gender norms on phone access. Women who report they alone decide on their health care report higher phone access than those whose husband made the decision and those who made joint decisions across both rural (53.3% vs 33.5%) and urban (68.7% vs 53.2%) settings. For contraception, women reporting joint decisions were more likely to report mobile phone access than those where the husband made the decisions. This held true in both rural (41.7% vs 34.3%) and urban (65.9% vs 52.9%) settings. Women saying no to justification of any reason to beat wife were more likely to have phone access and these levels were consistent across both urban and rural areas.

**Table 2:** Mobile phone ownership and use by variables for decision making and partner communication.

	Household phone ownership						Women’s access to phone						Women's ability to read SMS					
	Rural (Unweighted N = 198,248)			Urban (Unweighted N = 61,379)			Rural (Unweighted N = 34,078)			Urban (Unweighted N = 11,153)			Rural (Unweighted N = 14,029)			Urban (Unweighted N = 6,852)		
	%	95% CI		%	95% CI		%	95% CI		%	95% CI		%	95% CI		%	95% CI	
**Person who decides on respondent's health care**																		
Respondent alone	92.8	91.2	94.4	96.3	93.6	99.0	53.3	50.2	56.4	68.7	63.0	74.4	55	50.7	59.3	72.5	64.3	80.7
Respondent and husband/partner	92	91.4	92.6	96.9	96.1	97.7	43.1	41.9	44.3	66.2	64.0	68.4	62.4	60.6	64.2	80.9	78.7	83.1
Husband/partner alone	91.1	90.1	92.1	97.6	96.6	98.6	33.5	31.9	35.1	53.2	49.5	56.9	52.6	49.7	55.5	73.7	68.4	79.0
Other	97.4	95.2	99.6	96.6	92.7	100.0	38.4	31.9	44.9	52.9	40.4	65.4	65.3	55.1	75.5	83.5	72.3	94.7
Someone else	95.2	93.0	97.4	98.6	97.2	100.0	43.8	38.7	48.9	48.2	37.0	59.4	52.9	45.1	60.7	86.2	77.2	95.2
**Decision maker for using contraception**																		
Mainly respondent	90.9	89.5	92.3	96.5	94.9	98.1	39.9	35.0	44.8	58.2	48.8	67.6	58	50.2	65.8	79.4	70.0	88.8
Joint decision	92.5	92.1	92.9	97.7	97.3	98.1	41.7	40.1	43.3	65.9	63.2	68.6	64.5	62.1	66.9	79.3	75.6	83.0
Mainly husband, partner	89.6	88.4	90.8	94.8	92.6	97.0	34.3	30.2	38.4	52.9	43.7	62.1	50.3	43.2	57.4	64.6	51.9	77.3
Other	91.9	85.8	98.0	95.7	87.3	100.0	46.7	17.3	76.1	47.1	0	98.5	100	100.0	100.0	100	100.0	100.0
**Beating justified if wife goes out without telling husband**																		
No	92	91.4	92.6	97	96.2	97.8	42	41.0	43.0	63.4	61.4	65.4	61.9	60.3	63.5	80.4	78.0	82.8
Yes	91.6	90.6	92.6	97.1	95.7	98.5	40.5	38.7	42.3	60.6	56.9	64.3	52.1	49.4	54.8	72.6	68.5	76.7
**Beating justified if wife neglects the children**																		
No	91.8	91.2	92.4	96.7	95.9	97.5	42.4	41.4	43.4	61.9	59.7	64.1	61.8	60.2	63.4	80	77.5	82.5
Yes	92.1	91.3	92.9	97.7	96.9	98.5	40.1	38.5	41.7	64.5	61.4	67.6	54	51.5	56.5	75.2	71.7	78.7
**Beating justified if wife argues with husband**																		
No	92.3	91.7	92.9	96.8	96.0	97.6	43.1	42.1	44.1	63.6	61.6	65.6	63.5	61.9	65.1	80.1	77.7	82.5
Yes	91.1	90.1	92.1	97.4	96.4	98.4	38.5	36.9	40.1	60.1	56.6	63.6	49.3	46.8	51.8	73.2	69.3	77.1
**Beating justified if wife refuses to have sex with husband**																		
No	92	91.4	92.6	96.9	96.3	97.5	42.6	41.6	43.6	63.5	61.5	65.5	61.3	59.9	62.7	79.5	77.3	81.7
Yes	91.2	89.8	92.6	97.7	96.5	98.9	37	34.8	39.2	57.5	52.6	62.4	46.3	42.6	50.0	69.6	63.5	75.7
**Beating justified if wife doesn't cook food properly**																		
No	92.2	91.6	92.8	97.1	96.5	97.7	42.9	41.9	43.9	63.8	61.8	65.8	62.1	60.5	63.7	79.8	77.6	82.0
Yes	90.8	89.6	92.0	96.8	95.4	98.2	37.2	35.4	39.0	56.7	52.4	61.0	47.7	44.6	50.8	70.2	64.9	75.5

S2 and S3 Appendices present the levels of household mobile phone ownership and women’s phone access across the different indicators of health care seeking and key interventions.

### Multilevel models

Multilevel models were used to analyze the association of mobile phone access on the health care utilization from the MNCH continuum. The main effects, adjusted odds ratios(aOR) with 95% confidence intervals, of mobile phone access and the interaction effects of phone access with wealth, caste and education on four selected outcomes (Early ANC, Skilled attendance, Postnatal Care, Modern contraceptive use) are briefly summarized in Table 3. The full models for all outcomes are presented as S4 Appendix.

**Table 3:** Effect sizes for women’s mobile access for selected indicators of utilization of health care (adjusted odds ratios and 95% confidence intervals).

	Early ANC		Skilled attendance		Postnatal Care		Modern contraceptive use	
	Rural (N = 19,002)	Urban (N = 7,607)	Rural (N = 32,548)	Urban (N = 10,698)	Rural (N = 23,455)	Urban (N = 8,386)	Rural (N = 32548)	Urban (N = 10698)
**Main effect of mobile phone access**	1.19 (0.924,1.53)	0.52 (0.25,1.08)	1.16 (0.92,1.45)	1.77 (0.88,3.57)	0.97 (0.77,1.23)	2.04 (1.03,4.06)	1.1 (0.89,1.37)	1.83 (1.01,3.31)
**Interaction with wealth**								
Poorest	Ref	Ref	Ref	Ref	Ref	Ref	Ref	Ref
Poorer	0.95 (0.77,1.17)	2.49 (1.17,5.4)	0.86 (0.71,1.03)	0.65 (0.32,1.32)	0.82 (0.68,1)	0.76 (0.37,1.57)	0.92 (0.76,1.1)	0.56 (0.3,1.05)
Middle	1.02 (0.82,1.29)	2.15 (1.04,4.45)	0.9 (0.72,1.12)	0.54 (0.27,1.05)	1.13 (0.91,1.41)	00.55 (0.28,1.07)	0.96 (0.79,1.18)	0.66 (0.37,1.2)
Richer	0.86 (0.66,1.13)	2.11 (1.03,4.34)	1.12 (0.83,1.5)	0.89 (0.45,1.77)	1.36 (1.04,1.79)	0.75 (0.38,1.48)	0.93 (0.74,1.18)	0.61 (0.34,1.1)
Richest	1.04 (0.72,1.51)	2.51 (1.2,5.28)	1.44 (0.92,2.24)	0.69 (0.33,1.46)	1.41 (0.96,2.07)	0.69 (0.34,1.4)	1.11 (0.81,1.51)	0.73 (0.4,1.33)
**Interaction with caste **								
No education	Ref	Ref	Ref	Ref	Ref	Ref	Ref	Ref
Primary	1.07 (0.84,1.36)	1.17 (0.73,1.88)	1.01 (0.82,1.25)	1.27 (0.77,2.11)	1.19 (0.95,1.49)	0.94 (0.59,1.52)	0.94 (0.76,1.15)	0.59 (0.4,0.88)
Secondary	0.96 (0.79,1.17)	1.16 (0.79,1.7)	1.07 (0.89,1.28)	1.04 (0.68,1.58)	1.11 (0.92,1.33)	1.11 (0.11,1.62)	1.0 (0.84,1.19)	0.99 (0.72,1.36)
Higher	1.32 (0.89,1.96)	1.63 (0.89,2.97)	1.21 (0.71,2.09)	2.44 (1.03,5.78)	1.06 (0.7,1.62)	1.25 (0.66,2.37)	0.84 (0.58,1.21)	1.11 (0.67,1.86)
**Interaction with education**								
General category / No caste	Ref	Ref	Ref	Ref	Ref	Ref	Ref	Ref
Other Backward castes	0.98 (0.8,1.21)	0.79 (0.57,1.09)	0.97 (0.78,1.21)	0.96 (0.62,1.5)	1.0 (0.81,1.23)	0.82 (0.58,1.15)	0.86 (0.72,1.03)	0.95 (0.73,1.25)
Schedule castes	0.93 (0.73,1.17)	0.86 (0.57,1.28)	0.9 (0.7,1.15)	1.08 (0.63,1.85)	1.02 (0.8,1.29)	0.68 (0.44,1.03)	0.89 (0.73,1.1)	0.65 (0.46,0.91)
Schedule tribes	0.95 (0.75,1.21)	1.12 (0.7,1.8)	1.31 (1.03,1.67)	1.09 (0.59,2.02)	0.93 (0.74,1.18)	0.61 (0.37,1.00)	1.06 (0.86,1.31)	0.95 (0.62,1.44)

Mobile phone access is associated significantly with postnatal care (2.07, 1.03–4.07) and modern contraception 1.84 (1.01,3.31)) in the urban setting with no apparent influence in the rural population. Phone access is associated with greater use of early ANC for the richer quintiles compared to the poorest in the urban setting while no such trends are apparent for the rural population. Richer women showed lesser influence of phone access on postnatal care and modern contraceptive use compared to the poorest, but the findings did not reach statistical significance. Urban women with phone access with higher education were more likely to report skilled birth attendance (2.4,1.03–5.78) than women with no education, while urban women with primary education were less likely to report modern contraceptive use (0.59,0.4.0.88). Belonging to castes other than general category appeared to negatively influence the association of phone access with postnatal care and modern contraceptive use in the urban setting, but the associations were not statistically significant except for modern contraceptive use among scheduled caste women (0.65,0.46–0.91).

#### Decomposition analysis

**Fig 5:**
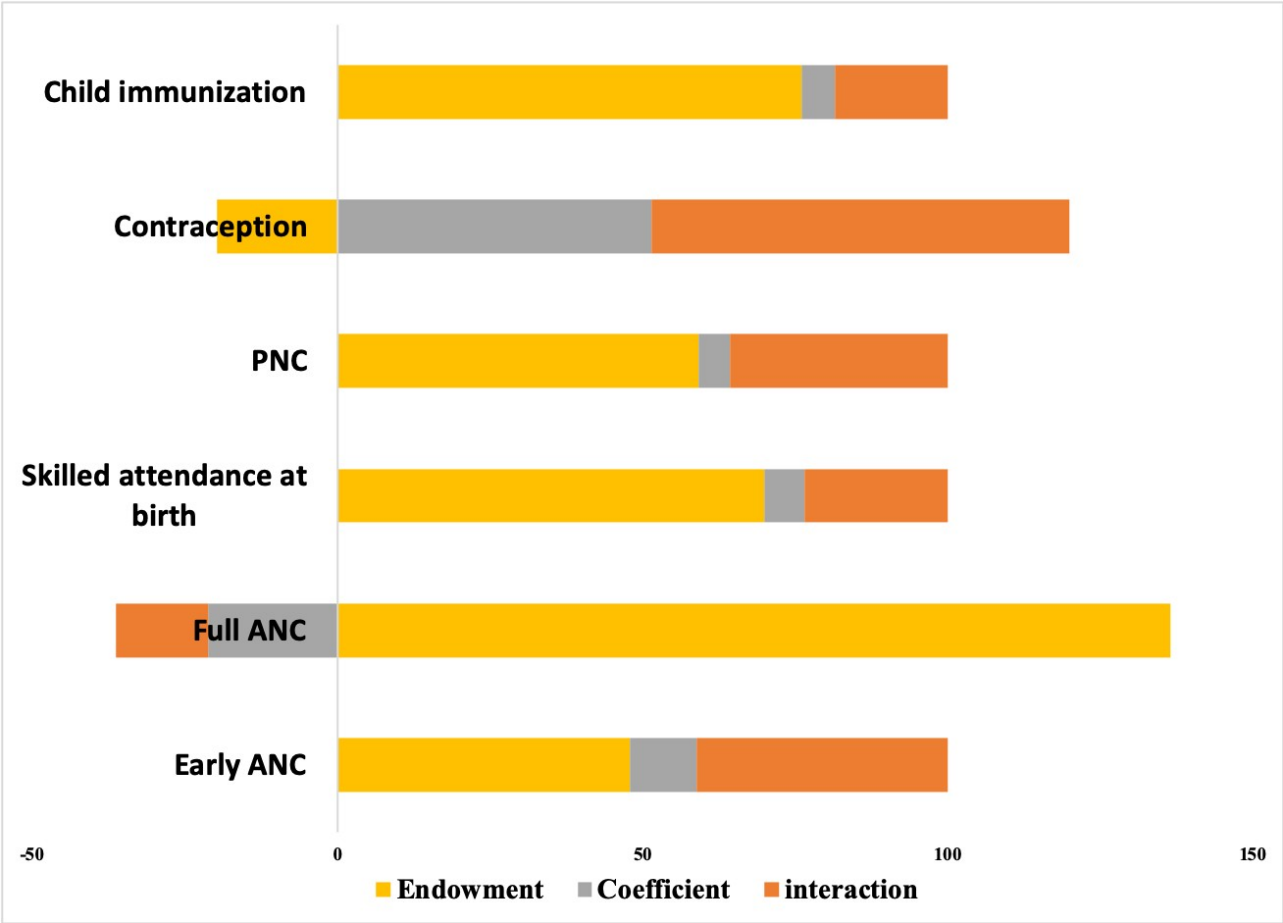
Blinder-Oaxaca decomposition plot explaining differences in health care utilization indicators between women with and without phone access.

The Oaxaca-Blinder decomposition analysis (Fig 5) quantifies the extent to which the gap in the prevalence of health outcomes between women with, and without, phone access can be explained. The prevalence gap is broken down into components: 1. Endowment gap—attributable to inherent differences between women with and without phone access in the magnitude of their background characteristics like wealth, education etc., which influence healthcare utilization (also called the “explained component”); 2. Coefficients—attributable solely to differences in their access to phone (also called the “unexplained component”); 3. Interaction–attributable to the interaction of the endowment and coefficient components. In Fig 5, the total gap is represented by the percentage attributed to each of the above components, with the individual components adding up to 100%. Bars to the left (negative values) suggest that an attribute decreases the gap in prevalence of an indicator, while bars to the right (positive values) suggest that it increases the gap. Using skilled birth attendance as an example (Fig 5), about 70% of the gap is explained by background characteristics, while 23.5% of the gap is due to the gap in phone access. Contraception with 68.4% has the highest coefficient component while full ANC has the lowest at -15.1% which means that phone access results in reducing the gap in utilization. Other indicators have significant endowment components indicating the utilization gap is more due to baseline characteristics rather than phone access.

## Discussion

Our analysis of NFHS data from India is the first to analyze the access for women of reproductive age and influence of mobile phones on health behaviors on a large nationally representative dataset within the context of reproductive, maternal and child health. Mobile phone ownership at the household level appears to be ubiquitous with very little urban rural divide. In contrast, the gap between household and women’s reported access to the mobile phones was 45% across states in India, and 49% in rural areas compared to 15% in urban areas. The wide variation across the various states of the Indian Union has also been observed in an analysis of the Intermedia Financial Inclusion Insights (FII) data from 2015–2016. Traditionally conservative states had wider gaps attributable in part to wealth and education and, primarily, to the cultural and social norms prevailing in these contexts. [23]

Inequalities in the distribution of the gap were observed by wealth, education, and caste, and these trends were similar across both rural and urban settings. Determinants associated with phone access appear to vary in direction and magnitude across states, possibly in line with the state’s maturity of phone access. Phone access was associated with improvements in health utilization in urban areas, including increased utilization of postnatal care and modern contraceptive use; however, a significant influence on health utilization in rural areas was not observed. A key finding is that the proxy variables for decision making and gender norms were not consistently associated with the health behaviors, possibly due to measurement issues.

Study findings highlight three key issues: 1) Gender inequality exists in mobile phone access; 2) The gender inequality in phone access by itself is associated with inequality due to state of residence, urban-rural, wealth, education and caste; and 3) The inequality in phone access is associated with inequality in prevalence of health behaviors. This results in a widening gulf with inequality building upon inequality–women suffer in terms of phone access; the poorest women suffer more, and the poorest women without phone access suffer the most in terms of health status. Urban living is the most important determinant of phone access in absolute terms and contributes to the attenuation of the inequities when compared to rural settings. Gender based inequality exists in other LMIC settings like Bangladesh in phone ownership, knowledge and awareness of mHealth programs, and intention to use mHealth services. [32,33] It has been a traditionally held view that women are technologically challenged and the reason for the existence of gender inequality (or digital divide) is that men are much better users of technology. [32] However, research shows that women are not “poor” users of mobile phones but are constrained by employment, education, social groupings and income. [8,23,34] When these constraints are adequately controlled, women are as active as, if not more than, men in the use of digital tools. [8] Our study supports the idea that the above reported barriers continue to hinder women’s effective access of mobile phone coverage.

The socio-economic gradient in the gender inequality may be explained in part by the cost of mobile phone ownership including the cost of a handset and recurring charges for a phone connection. Cost of handsets and service have been reported as leading barriers to phone ownership by women elsewhere. [10] With the cost of ownership falling dramatically and expected to continue over the coming years, women’s access to phones is likely increase. However, differentials in that increase in phone access by socio-demographic characteristics are likely to persist. Elsewhere in Kenya and Bangladesh, similar trends have been observed with gender across education levels. [32,33,35] Low literacy and difficulty with non-English complex language interfaces have also been reported to be an impediment to use among women in many parts of the world. [36] Conversely, education empowers women and results in increased ownership of a mobile phone, as mobile phone capabilities are directly related to education. [23]

The poorest and most marginalized women are the most likely to have higher health morbidity and mortality. [37] Mobile phones have the potential to reduce health disparities, especially those attributable to poor utilization of health services due to high cost, far distance, and inadequate health infrastructure. [38] However, by not actively considering gaps in mobile phone access, the rapid expansion of maternal mHealth applications targeting clients, such as appointment reminders, behavior change messages, or supportive care, is likely to exacerbate already existing inequalities among women in accessing care. [17, 39] Addressing the gap in mobile phone access has the potential to improve the health of millions of women. GSMA reports that 84% of women want better health care-related information and 39% of women express an interest in receiving the information through mobile phones. [40] Mobile phone access for women on its own will not change health status but will need a host of converging factors, such as health systems availability and improved quality of care, to achieve the health outcomes. [33]

### Limitations

Our conceptual approach and framework assumes a linear approach, while, in reality, the factors described interact with one another in a complex manner. There are potential data limitations to be borne in mind while interpreting our findings. The first is the issue of causality since the cross-sectional nature of survey data makes it impossible to determine whether phone access preceded health behaviors. Secondly, there is a need to understand the possibility of selection bias where the most vulnerable population groups (like nomadic groups and refugees) are either missing from the sampling frame or more likely to be unavailable for interviewing. These are also groups likely to have the least levels of mobile penetrance and poor health outcomes caused by a lack of access to the formal health system. Third, the indicators used to assess phone access and use, query respondents as to whether “[they] have any mobile phone that you yourself use?”. This measure is limited in that it combines access (“do you have any mobile phone”) with digital literacy (“that you yourself use”). While a second question asks, “Are you able to read text (SMS) messages?”, it relies on reported literacy and phone capabilities without observing the behavior. Beyond the limitations of these questions, we note that an information bias may exist when women report phone access due to social desirability which may vary across different groups. Also, the survey may not capture the concepts of gender norms and decision making & autonomy adequately to address the pathway from mobile phone use to health behavior.

Sample size considerations in multilevel analysis are usually related to the sample size at the various levels, i.e. number of states and districts. Studies suggest that the standard errors and the variance components tend to be underestimated when the number of higher-level units is less than 30. [41] Therefore, the relatively small number of higher level units in this paper (*n* = 29 states) implies that the state-level random variances and, hence, the standard errors may have been underestimated. Finally, the decomposition methods do not consider the hierarchical nature of the data and effects of group membership on the level of the outcomes between the groups with and without phone access.

## Conclusions

The increase in access to mobile phones globally has been characterized by inequalities by gender, geographic areas, and sociodemographic characteristics. For the success of large digital health programs, near complete coverage of the target population is needed while our study findings reveal a large population of women of reproductive age in India without phone access. Unless efforts are made to improve access to phones among women, inequalities in use of health services and adoption of health behaviors are likely to persist. Efforts to link phone access to improved care seeking, and practices suggests that while phone access was associated with improvements in urban health including modern contraceptive use and increased postnatal care, a significant influence on rural health was not observed. Further research is warranted in understanding the differential effect of phone access on health outcomes and exploration of women’s autonomy and decision making on health care seeking.

## Supporting information

S1 Appendix Mobile phone ownership and use by demographic characteristics. (DOCX)

S2 Appendix Mobile phone ownership and use by healthcare utilization. (DOCX)

S3 Appendix Mobile phone ownership and use across levels of key intervention indicators. (DOCX)

S4 Appendix Multilevel models for association of women’s phone access with maternal and child outcomes. (DOCX)
