# Supplementary material for: Does having a mobile phone matter? Linking phone access among women to health in India: An exploratory analysis of the National Family Health Survey
Source: PLoS One. 2020 Jul 20;15(7):e0236078. doi: 10.1371/journal.pone.0236078 (PMC7371204; doi:10.1371/journal.pone.0236078)
Supplement: S1 Appendix — (DOCX) [file pone.0236078.s001.docx]

**S1Table B1. Mobile phone ownership and use by Demographic Characteristics**

|  | **Household ownership** | | | | | | **Women report access to phone** | | | | | | **Women's ability to read SMS** | | | | | |
| --- | --- | --- | --- | --- | --- | --- | --- | --- | --- | --- | --- | --- | --- | --- | --- | --- | --- | --- |
|  | **Rural (Unweighted N = 198,248)** | | | **Urban (Unweighted N =61,379)** | | | **Rural  (Unweighted N =34,078)** | | | **Urban (Unweighted N = 11,153)** | | | **Rural (Unweighted N =14,029)** | | | **Urban (Unweighted N = 6,852)** | | |
|  | **%** | **95% CI** | | **%** | **95% CI** | | **%** | **95% CI** | | **%** | **95% CI** | | **%** | **95% CI** | | **%** | **95% CI** | |
| **Religion** |  |  |  |  |  |  |  |  |  |  |  |  |  |  |  |  |  |  |
| Christian | 86.8 | 85.2 | 88.4 | 97.9 | 96.3 | 99.5 | 50.6 | 44.9 | 56.3 | 85.7 | 78.6 | 92.8 | 81.7 | 76.2 | 87.2 | 91.2 | 85.7 | 96.7 |
| Hindu | 91.00 | 90.8 | 91.2 | 97.2 | 97.0 | 97.4 | 41 | 40.0 | 42.0 | 64.3 | 62.1 | 66.5 | 59.5 | 57.9 | 61.1 | 79 | 76.5 | 81.5 |
| Muslim | 92.5 | 91.9 | 93.1 | 96.3 | 95.7 | 96.9 | 44 | 41.6 | 46.4 | 54.1 | 50.6 | 57.6 | 49.9 | 46.2 | 53.6 | 73.1 | 69.2 | 77.0 |
| Other religion | 90.3 | 89.1 | 91.5 | 97.9 | 96.7 | 99.1 | 40.9 | 35.6 | 46.2 | 67.9 | 58.9 | 76.9 | 81.1 | 76.2 | 86.0 | 83.8 | 73.8 | 93.8 |
| **Wealth index** |  |  |  |  |  |  |  |  |  |  |  |  |  |  |  |  |  |  |
| Poorest | 78.7 | 78.1 | 79.3 | 73.7 | 70.8 | 76.6 | 27.8 | 26.4 | 29.2 | 26.3 | 19.0 | 33.6 | 25.6 | 22.9 | 28.3 | 17.8 | 4.3 | 31.3 |
| Poorer | 95.2 | 94.8 | 95.6 | 90.2 | 88.6 | 91.8 | 36.4 | 34.8 | 38.0 | 29.2 | 24.3 | 34.1 | 47.8 | 44.9 | 50.7 | 38.3 | 29.1 | 47.5 |
| Middle | 98.4 | 98.2 | 98.6 | 97.4 | 96.8 | 98.0 | 45.7 | 43.7 | 47.7 | 47.3 | 43.2 | 51.4 | 65.4 | 62.7 | 68.1 | 58.5 | 52.4 | 64.6 |
| Richer | 99.5 | 99.3 | 99.7 | 99.2 | 99.0 | 99.4 | 61.3 | 58.8 | 63.8 | 63.1 | 59.6 | 66.6 | 79.5 | 77.0 | 82.0 | 72.8 | 68.7 | 76.9 |
| Richest | 99.7 | 99.5 | 99.9 | 99.6 | 99.4 | 99.8 | 74.8 | 71.9 | 77.7 | 82 | 79.8 | 84.2 | 91.4 | 89.6 | 93.2 | 91.9 | 89.5 | 94.3 |
| **Social classification** |  |  |  |  |  |  |  |  |  |  |  |  |  |  |  |  |  |  |
| General category / No caste | 94.7 | 94.3 | 95.1 | 98 | 97.6 | 98.4 | 53.3 | 50.9 | 55.7 | 68.5 | 64.8 | 72.2 | 72.1 | 69.4 | 74.8 | 84.3 | 79.8 | 88.8 |
| Other Backward castes | 93.9 | 93.7 | 94.1 | 97.6 | 97.2 | 98.0 | 44.2 | 42.8 | 45.6 | 62.4 | 59.9 | 64.9 | 57 | 55.0 | 59.0 | 75.8 | 73.1 | 78.5 |
| Schedule castes | 89.8 | 89.2 | 90.4 | 95.3 | 94.5 | 96.1 | 36.5 | 34.7 | 38.3 | 55.2 | 51.3 | 59.1 | 49.8 | 46.7 | 52.9 | 72.5 | 67.8 | 77.2 |
| Schedule tribes | 79.3 | 78.5 | 80.1 | 92.5 | 90.5 | 94.5 | 26.2 | 24.2 | 28.2 | 53.7 | 46.1 | 61.3 | 58.7 | 54.4 | 63.0 | 71.4 | 61.2 | 81.6 |
| **Age (5-year groups)** |  |  |  |  |  |  |  |  |  |  |  |  |  |  |  |  |  |  |
| 15-19 | 91.2 | 90.0 | 92.4 | 95.1 | 92.4 | 97.8 | 33.4 | 28.7 | 38.1 | 45.3 | 32.4 | 58.2 | 72.9 | 65.5 | 80.3 | 72.4 | 54.8 | 90.0 |
| 20-24 | 92.7 | 92.3 | 93.1 | 97.2 | 96.8 | 97.6 | 41.4 | 39.8 | 43.0 | 55.9 | 52.0 | 59.8 | 65.5 | 63.1 | 67.9 | 74.7 | 69.8 | 79.6 |
| 25-29 | 91.9 | 91.5 | 92.3 | 97.2 | 96.8 | 97.6 | 43.7 | 42.3 | 45.1 | 65.3 | 62.8 | 67.8 | 60.7 | 58.5 | 62.9 | 79.2 | 76.1 | 82.3 |
| 30-34 | 89.2 | 88.6 | 89.8 | 97 | 96.4 | 97.6 | 42.3 | 40.3 | 44.3 | 69.1 | 65.8 | 72.4 | 50.6 | 47.5 | 53.7 | 82.5 | 79.6 | 85.4 |
| 35-39 | 85.7 | 84.7 | 86.7 | 96.3 | 95.3 | 97.3 | 36.9 | 34.0 | 39.8 | 63.5 | 58.0 | 69.0 | 42.4 | 37.5 | 47.3 | 76.1 | 70.2 | 82.0 |
| 40-44 | 83.9 | 82.1 | 85.7 | 95.6 | 93.6 | 97.6 | 28.9 | 23.6 | 34.2 | 54.2 | 43.4 | 65.0 | 19.6 | 12.7 | 26.5 | 74.4 | 61.9 | 86.9 |
| 45-49 | 81.4 | 78.5 | 84.3 | 87.9 | 75.2 | 100.6 | 22.3 | 13.7 | 30.9 | 41.5 | 14.6 | 68.4 | 31.2 | 5.5 | 56.9 | 72.1 | 32.7 | 111.5 |
| **Parity** |  |  |  |  |  |  |  |  |  |  |  |  |  |  |  |  |  |  |
| More than 2 | 88.2 | 87.8 | 88.6 | 95 | 94.4 | 95.6 | 33.8 | 32.4 | 35.2 | 47.0 | 43.5 | 50.5 | 37.6 | 35.1 | 40.1 | 59.5 | 54.8 | 64.2 |
| 2 children | 92.8 | 92.4 | 93.2 | 97.6 | 97.2 | 98.0 | 44.9 | 43.3 | 46.5 | 64.8 | 61.9 | 67.7 | 65.8 | 63.6 | 68.0 | 77.7 | 74.0 | 81.4 |
| One child | 93.8 | 93.4 | 94.2 | 98.1 | 97.7 | 98.5 | 50.4 | 48.8 | 52.0 | 73.0 | 70.5 | 75.5 | 75.5 | 73.7 | 77.3 | 89.4 | 87.6 | 91.2 |
| **Education** |  |  |  |  |  |  |  |  |  |  |  |  |  |  |  |  |  |  |
| No education | 85.00 | 84.6 | 85.4 | 92.2 | 91.2 | 93.2 | 26.5 | 25.1 | 27.9 | 30.7 | 27.0 | 34.4 | 4.3 | 3.1 | 5.5 | 7.8 | 3.9 | 11.7 |
| Primary | 89.7 | 89.1 | 90.3 | 95.1 | 94.1 | 96.1 | 34.7 | 32.5 | 36.9 | 38.4 | 33.1 | 43.7 | 34.6 | 30.1 | 39.1 | 36.4 | 27.0 | 45.8 |
| Secondary | 95.5 | 95.3 | 95.7 | 98 | 97.6 | 98.4 | 49.2 | 47.8 | 50.6 | 64.7 | 62.2 | 67.2 | 75.5 | 73.9 | 77.1 | 79.8 | 77.1 | 82.5 |
| Higher | 98.9 | 98.7 | 99.1 | 99.4 | 99.2 | 99.6 | 81.8 | 79.3 | 84.3 | 91.7 | 89.9 | 93.5 | 98 | 97.0 | 99.0 | 97.7 | 94.8 | 100.0 |
| **Husband education level** |  |  |  |  |  |  |  |  |  |  |  |  |  |  |  |  |  |  |
| Don't know | 91.2 | 83.9 | 98.5 | 91.9 | 79.9 | 103.9 | 36.1 | 20.0 | 52.2 | 42.2 | 18.1 | 66.3 | 27.5 | 6.7 | 48.3 | 81.0 | 53.8 | 100.0 |
| No education | 82.7 | 81.3 | 84.1 | 86.3 | 82.2 | 90.4 | 27.6 | 25.8 | 29.4 | 33.2 | 28.5 | 37.9 | 19.7 | 16.6 | 22.8 | 32.8 | 24.6 | 41.0 |
| Primary | 89.1 | 87.7 | 90.5 | 94.6 | 92.2 | 97.0 | 30.1 | 27.9 | 32.3 | 43.4 | 38.1 | 48.7 | 36.3 | 32.0 | 40.6 | 54.1 | 45.5 | 62.7 |
| Secondary | 95.00 | 94.4 | 95.6 | 98.6 | 98.0 | 99.2 | 45.8 | 44.6 | 47.0 | 62.1 | 59.6 | 64.6 | 64.1 | 62.3 | 65.9 | 76.0 | 72.9 | 79.1 |
| Higher | 99.1 | 98.7 | 99.5 | 99.2 | 98.6 | 99.8 | 66.5 | 64.0 | 69.0 | 87.0 | 84.8 | 89.2 | 89.4 | 87.4 | 91.4 | 95.6 | 94.0 | 97.2 |
| **Frequency of listening to multimedia** |  |  |  |  |  |  |  |  |  |  |  |  |  |  |  |  |  |  |
| Almost every day | 96.4 | 96.2 | 96.6 | 98.2 | 98.0 | 98.4 | 51.7 | 50.3 | 53.1 | 67.9 | 65.9 | 69.9 | 73.7 | 72.1 | 75.3 | 81.9 | 79.7 | 84.1 |
| At least once a week | 90.9 | 90.3 | 91.5 | 94.6 | 93.2 | 96.0 | 36.2 | 33.8 | 38.6 | 54.4 | 48.1 | 60.7 | 61.1 | 57.2 | 65.0 | 68.1 | 59.9 | 76.3 |
| Less than a week | 90.2 | 89.6 | 90.8 | 94.5 | 93.1 | 95.9 | 36.6 | 33.9 | 39.3 | 45.3 | 38.0 | 52.6 | 56 | 51.3 | 60.7 | 59.8 | 49.2 | 70.4 |
| Not at all | 84.6 | 84.2 | 85.0 | 89.6 | 88.0 | 91.2 | 30.4 | 29.0 | 31.8 | 31.6 | 26.3 | 36.9 | 22.7 | 20.3 | 25.1 | 36.4 | 26.8 | 46.0 |
