# Supplementary material for: Does having a mobile phone matter? Linking phone access among women to health in India: An exploratory analysis of the National Family Health Survey
Source: PLoS One. 2020 Jul 20;15(7):e0236078. doi: 10.1371/journal.pone.0236078 (PMC7371204; doi:10.1371/journal.pone.0236078)
Supplement: S2 Appendix — (DOCX) [file pone.0236078.s002.docx]

**S2Table B2. Mobile phone ownership and use by healthcare utilization**

|  | **Household ownership** | | | | | | **Women report acces to phone** | | | | | | **Women's ability to read SMS** | | | | | |
| --- | --- | --- | --- | --- | --- | --- | --- | --- | --- | --- | --- | --- | --- | --- | --- | --- | --- | --- |
|  | **Rural** | | | **Urban** | | | **Rural** | | | **Urban** | | | **Rural** | | | **Urban** | | |
|  | **%** | **95% CI** | | **%** | **95% CI** | | **%** | **95% CI** | | **%** | **95% CI** | | **%** | **95% CI** | | **%** | **95% CI** | |
| **Pregnancy registered** |  |  |  |  |  |  |  |  |  |  |  |  |  |  |  |  |  |  |
| No | 88.4 | 87.8 | 89.0 | 96.7 | 96.1 | 97.3 | 38.6 | 36.4 | 40.8 | 59.7 | 55.2 | 64.2 | 43.6 | 40.1 | 47.1 | 75.3 | 69.2 | 81.4 |
| Yes | 91.9 | 91.7 | 92.1 | 97.4 | 97.2 | 97.6 | 44 | 43.0 | 45.0 | 66.2 | 64.4 | 68.0 | 64 | 62.6 | 65.4 | 81.4 | 79.6 | 83.2 |
| **Receive Mother and Child Protection Card after registration** |  |  |  |  |  |  |  |  |  |  |  |  |  |  |  |  |  |  |
| No | 90.1 | 89.5 | 90.7 | 97.8 | 97.2 | 98.4 | 43.4 | 40.7 | 46.1 | 69.3 | 64.8 | 73.8 | 52.2 | 47.9 | 56.5 | 82.9 | 78.8 | 87.0 |
| Yes | 92.1 | 91.9 | 92.3 | 97.3 | 97.1 | 97.5 | 44.1 | 43.1 | 45.1 | 65.8 | 64.0 | 67.6 | 65.2 | 63.8 | 66.6 | 81.2 | 79.4 | 83.0 |
| **Child's father present during any antenatal visit** |  |  |  |  |  |  |  |  |  |  |  |  |  |  |  |  |  |  |
| No | 91.4 | 91.0 | 91.8 | 96 | 95.2 | 96.8 | 45.8 | 43.8 | 47.8 | 55 | 50.1 | 59.9 | 56.9 | 54.0 | 59.8 | 72.8 | 66.7 | 78.9 |
| Yes | 92.7 | 92.5 | 92.9 | 97.8 | 97.6 | 98.0 | 45 | 44.0 | 46.0 | 68.8 | 67.0 | 70.6 | 68.3 | 66.7 | 69.9 | 83 | 81.2 | 84.8 |
| **Ultra sound test** |  |  |  |  |  |  |  |  |  |  |  |  |  |  |  |  |  |  |
| No | 86.2 | 85.8 | 86.6 | 91.6 | 90.6 | 92.6 | 31.4 | 30.2 | 32.6 | 36.9 | 33.2 | 40.6 | 35.7 | 33.3 | 38.1 | 54 | 47.3 | 60.7 |
| Yes | 94.6 | 94.4 | 94.8 | 98 | 97.8 | 98.2 | 48.4 | 47.2 | 49.6 | 67.4 | 65.4 | 69.4 | 69 | 67.4 | 70.6 | 80.8 | 78.6 | 83.0 |
| **During last 3 months of pregnancy met ANM, LHV, ASHA, anganwadi worker or other** |  |  |  |  |  |  |  |  |  |  |  |  |  |  |  |  |  |  |
| No | 91 | 90.8 | 91.2 | 97.6 | 97.4 | 97.8 | 42.8 | 41.6 | 44.0 | 66.5 | 64.3 | 68.7 | 58.5 | 56.5 | 60.5 | 82.3 | 80.1 | 84.5 |
| Yes | 91.7 | 91.5 | 91.9 | 96.9 | 96.5 | 97.3 | 43.7 | 42.5 | 44.9 | 63.6 | 61.2 | 66.0 | 63.8 | 62.0 | 65.6 | 78.1 | 75.6 | 80.6 |
| **Receive advice on family planning** |  |  |  |  |  |  |  |  |  |  |  |  |  |  |  |  |  |  |
| No | 90.9 | 90.5 | 91.3 | 96.8 | 96.0 | 97.6 | 41.6 | 39.6 | 43.6 | 62.3 | 57.8 | 66.8 | 54.9 | 52.0 | 57.8 | 68.9 | 62.8 | 75.0 |
| Yes | 92 | 91.6 | 92.4 | 96.9 | 96.5 | 97.3 | 44.6 | 43.2 | 46.0 | 64 | 61.3 | 66.7 | 67.5 | 65.5 | 69.5 | 80.8 | 78.3 | 83.3 |
| **Receive financial assistance for delivery care** |  |  |  |  |  |  |  |  |  |  |  |  |  |  |  |  |  |  |
| No | 94.3 | 94.1 | 94.5 | 98.2 | 98.0 | 98.4 | 47.7 | 46.3 | 49.1 | 70.1 | 68.1 | 72.1 | 72.4 | 70.6 | 74.2 | 84.9 | 82.9 | 86.9 |
| Yes | 91.7 | 91.5 | 91.9 | 96.9 | 96.5 | 97.3 | 44.6 | 43.2 | 46.0 | 61.9 | 59.2 | 64.6 | 58.3 | 56.3 | 60.3 | 74.4 | 71.3 | 77.5 |
