# Supplementary material for: Does having a mobile phone matter? Linking phone access among women to health in India: An exploratory analysis of the National Family Health Survey
Source: PLoS One. 2020 Jul 20;15(7):e0236078. doi: 10.1371/journal.pone.0236078 (PMC7371204; doi:10.1371/journal.pone.0236078)
Supplement: S3 Appendix — (DOCX) [file pone.0236078.s003.docx]

**S3 Table B3. Mobile phone ownership and use across levels of key intervention indicators**

|  | **Household ownership** | | | | | | **Women's access to phone** | | | | | | **Women's ability to read SMS** | | | | | |
| --- | --- | --- | --- | --- | --- | --- | --- | --- | --- | --- | --- | --- | --- | --- | --- | --- | --- | --- |
|  | **Rural** | | | **Urban** | | | **Rural** | | | **Urban** | | | **Rural** | | | **Urban** | | |
|  | **%** | **95% CI** | | **%** | **95% CI** | | **%** | **95% CI** | | **%** | **95% CI** | | **%** | **95% CI** | | **%** | **95% CI** | |
| **Early check up** |  |  |  |  |  |  |  |  |  |  |  |  |  |  |  |  |  |  |
| No | 90.6 | 90.2 | 91.0 | 96.7 | 96.1 | 97.3 | 39.7 | 38.1 | 41.3 | 58.2 | 54.3 | 62.1 | 56.7 | 54.0 | 59.4 | 76.3 | 72.4 | 80.2 |
| Yes | 93.3 | 93.1 | 93.5 | 97.9 | 97.7 | 98.1 | 47.6 | 46.4 | 48.8 | 69.5 | 67.7 | 71.3 | 69.4 | 67.8 | 71.0 | 83.2 | 81.2 | 85.2 |
| **Number of ANC** |  |  |  |  |  |  |  |  |  |  |  |  |  |  |  |  |  |  |
| At least 4 visits | 93 | 92.6 | 93.4 | 97.8 | 97.4 | 98.2 | 46.3 | 44.9 | 47.7 | 67.9 | 65.5 | 70.3 | 70.9 | 69.1 | 72.7 | 82.1 | 79.7 | 84.5 |
| Less than 4 visits | 91.9 | 91.7 | 92.1 | 97.3 | 96.9 | 97.7 | 44.1 | 42.9 | 45.3 | 65.5 | 63.0 | 68.0 | 60.9 | 58.9 | 62.9 | 81.4 | 79.0 | 83.8 |
| No antenatal visits | 87 | 86.4 | 87.6 | 94.4 | 93.4 | 95.4 | 34.6 | 32.8 | 36.4 | 49.8 | 44.5 | 55.1 | 34.7 | 31.6 | 37.8 | 63.1 | 55.3 | 70.9 |
| **Tetanus vaccination** |  |  |  |  |  |  |  |  |  |  |  |  |  |  |  |  |  |  |
| No | 89.9 | 89.5 | 90.3 | 96.1 | 95.7 | 96.5 | 37.4 | 36.0 | 38.8 | 56.4 | 53.1 | 59.7 | 52.8 | 50.6 | 55.0 | 71 | 66.7 | 75.3 |
| Yes | 91.9 | 91.7 | 92.1 | 97.5 | 97.3 | 97.7 | 44.1 | 43.1 | 45.1 | 65.6 | 63.8 | 67.4 | 62.4 | 61.0 | 63.8 | 81.4 | 79.6 | 83.2 |
| **Iron folic acid (IFA) for 100 days or more during pregnancy** |  |  |  |  |  |  |  |  |  |  |  |  |  |  |  |  |  |  |
| No | 89.8 | 89.4 | 90.2 | 95.9 | 95.3 | 96.5 | 38.2 | 36.6 | 39.8 | 51.6 | 47.7 | 55.5 | 42.7 | 40.2 | 45.2 | 74.1 | 69.8 | 78.4 |
| Yes | 92 | 91.8 | 92.2 | 97.6 | 97.4 | 97.8 | 45.2 | 44.2 | 46.2 | 68.7 | 66.9 | 70.5 | 67.5 | 66.1 | 68.9 | 81.8 | 80.0 | 83.6 |
| **Full ANC (Early registration + Tetanus + IFA)** |  |  |  |  |  |  |  |  |  |  |  |  |  |  |  |  |  |  |
| No | 90.5 | 90.3 | 90.7 | 96.6 | 96.2 | 97.0 | 39.9 | 38.9 | 40.9 | 59.1 | 56.9 | 61.3 | 54 | 52.4 | 55.6 | 75.8 | 73.4 | 78.2 |
| Yes | 93.1 | 92.7 | 93.5 | 97.9 | 97.5 | 98.3 | 46.6 | 45.0 | 48.2 | 69.2 | 66.8 | 71.6 | 72.6 | 70.6 | 74.6 | 82.5 | 80.0 | 85.0 |
| **Skilled birth attendance** |  |  |  |  |  |  |  |  |  |  |  |  |  |  |  |  |  |  |
| No | 85.7 | 85.1 | 86.3 | 92.3 | 91.1 | 93.5 | 31.8 | 30.2 | 33.4 | 37.8 | 33.3 | 42.3 | 37.4 | 34.3 | 40.5 | 47 | 39.6 | 54.4 |
| Yes | 92.6 | 92.4 | 92.8 | 97.6 | 97.4 | 97.8 | 44.2 | 43.2 | 45.2 | 65.3 | 63.3 | 67.3 | 63.4 | 62.0 | 64.8 | 80.3 | 78.1 | 82.5 |
| **Facility delivery** |  |  |  |  |  |  |  |  |  |  |  |  |  |  |  |  |  |  |
| No | 86 | 85.6 | 86.4 | 92.3 | 91.1 | 93.5 | 31.9 | 30.3 | 33.5 | 37.7 | 33.6 | 41.8 | 36.8 | 33.9 | 39.7 | 48.7 | 41.8 | 55.6 |
| Yes | 92.8 | 92.6 | 93.0 | 97.6 | 97.4 | 97.8 | 44.5 | 43.5 | 45.5 | 65.6 | 63.6 | 67.6 | 64 | 62.6 | 65.4 | 80.4 | 78.2 | 82.6 |
| **Birth delivered by caesarean section** |  |  |  |  |  |  |  |  |  |  |  |  |  |  |  |  |  |  |
| No | 90.4 | 90.2 | 90.6 | 96.4 | 96.0 | 96.8 | 39.4 | 38.4 | 40.4 | 57.9 | 55.7 | 60.1 | 55.7 | 54.1 | 57.3 | 75.8 | 73.4 | 78.2 |
| Yes | 95.7 | 95.3 | 96.1 | 98.8 | 98.6 | 99.0 | 55.2 | 52.7 | 57.7 | 73.9 | 71.0 | 76.8 | 74.5 | 71.8 | 77.2 | 83.3 | 79.2 | 87.4 |
| **Received Postnatal care** |  |  |  |  |  |  |  |  |  |  |  |  |  |  |  |  |  |  |
| No | 91.5 | 91.3 | 91.7 | 97.4 | 97.2 | 97.6 | 43.5 | 42.7 | 44.3 | 65.6 | 64.0 | 67.2 | 61.7 | 60.3 | 63.1 | 80.7 | 78.9 | 82.5 |
| Yes | 88.3 | 87.1 | 89.5 | 94.1 | 91.9 | 96.3 | 35.9 | 31.6 | 40.2 | 54.5 | 43.9 | 65.1 | 53.4 | 45.6 | 61.2 | 75.6 | 65.0 | 86.2 |
| **Use of modern contraceptive method** |  |  |  |  |  |  |  |  |  |  |  |  |  |  |  |  |  |  |
| No | 90.7 | 90.5 | 90.9 | 96.7 | 96.3 | 97.1 | 42 | 41.0 | 43.0 | 61.8 | 59.4 | 64.2 | 57.4 | 55.8 | 59.0 | 78.8 | 76.6 | 81.0 |
| Yes | 92 | 91.6 | 92.4 | 97.5 | 97.1 | 97.9 | 40.9 | 39.3 | 42.5 | 63.9 | 61.2 | 66.6 | 62.8 | 60.4 | 65.2 | 77.9 | 74.2 | 81.6 |
| **Unmet need for contraception** |  |  |  |  |  |  |  |  |  |  |  |  |  |  |  |  |  |  |
| No | 91.1 | 90.9 | 91.3 | 97 | 96.6 | 97.4 | 39.8 | 38.8 | 40.8 | 63.1 | 61.1 | 65.1 | 60.5 | 58.9 | 62.1 | 78.5 | 76.0 | 81.0 |
| Yes | 91.1 | 90.7 | 91.5 | 97.2 | 96.8 | 97.6 | 46.4 | 44.6 | 48.2 | 61.5 | 57.8 | 65.2 | 56.9 | 54.4 | 59.4 | 77.8 | 74.3 | 81.3 |
