# Supplementary material for: Does having a mobile phone matter? Linking phone access among women to health in India: An exploratory analysis of the National Family Health Survey
Source: PLoS One. 2020 Jul 20;15(7):e0236078. doi: 10.1371/journal.pone.0236078 (PMC7371204; doi:10.1371/journal.pone.0236078)
Supplement: S4 Appendix — (DOCX) [file pone.0236078.s004.docx]

**S4 Table B4. Multilevel models for association of mobile phone access with ANC indicators**

|  | **Full ANC** | | | | | | | | **Early ANC** | | | | | | | |
| --- | --- | --- | --- | --- | --- | --- | --- | --- | --- | --- | --- | --- | --- | --- | --- | --- |
|  | **Rural** | | | | **Urban** | | | | **Rural** | | | | **Urban** | | | |
|  | **OR** | **95% CI** | | **p value** | **OR** | **95% CI** | | **p value** | **OR** | **95% CI** | | **p value** | **OR** | **95% CI** | | **p value** |
|  | **N=19,002** | | | | **N=7,607** | | | | **N=19,002** | | | | **N=7,607** | | | |
| **Mobile phone access** |  |  |  |  |  |  |  |  |  |  |  |  |  |  |  |  |
| No mobile phone access | Ref |  |  |  | Ref |  |  |  | Ref |  |  |  | Ref |  |  |  |
| Has access | 1.21 | 0.96 | 1.52 | 0.11 | 0.9 | 0.46 | 1.75 | 0.75 | 1.2 | 0.94 | 1.55 | 0.15 | 0.51 | 0.24 | 1.07 | 0.08 |
| **Multimedia** |  |  |  |  |  |  |  |  |  |  |  |  |  |  |  |  |
| No exposure | Ref |  |  |  | Ref |  |  |  | Ref |  |  |  | Ref |  |  |  |
| Some exposure | 1.23 | 1.14 | 1.34 | 0.00 | 1.31 | 1.11 | 1.55 | 0.00 | 1.13 | 1.04 | 1.24 | 0.01 | 1.33 | 1.09 | 1.63 | 0.00 |
| **Wealth index** |  |  |  |  |  |  |  |  |  |  |  |  |  |  |  |  |
| Poorest | Ref |  |  |  | Ref |  |  |  | Ref |  |  |  | Ref |  |  |  |
| Poorer | 1.27 | 1.14 | 1.43 | 0.00 | 1.05 | 0.73 | 1.49 | 0.81 | 1.04 | 0.92 | 1.18 | 0.48 | 0.56 | 0.36 | 0.86 | 0.01 |
| Middle | 1.69 | 1.48 | 1.93 | 0.00 | 1.47 | 1.04 | 2.08 | 0.03 | 1.35 | 1.16 | 1.57 | 0.00 | 0.64 | 0.41 | 0.98 | 0.04 |
| Richer | 1.88 | 1.59 | 2.24 | 0.00 | 1.44 | 1.01 | 2.06 | 0.04 | 1.6 | 1.3 | 1.97 | 0.00 | 0.68 | 0.44 | 1.06 | 0.09 |
| Richest | 2.06 | 1.6 | 2.64 | 0.00 | 1.63 | 1.11 | 2.39 | 0.01 | 1.58 | 1.16 | 2.14 | 0.00 | 0.75 | 0.47 | 1.2 | 0.23 |
| **Age** |  |  |  |  |  |  |  |  |  |  |  |  |  |  |  |  |
| 15-24 | Ref |  |  |  | Ref |  |  |  | Ref |  |  |  | Ref |  |  |  |
| 25-34 | 1.35 | 1.25 | 1.45 | 0.00 | 1.28 | 1.14 | 1.44 | 0.00 | 1.11 | 1.02 | 1.21 | 0.02 | 1.15 | 0.99 | 1.33 | 0.08 |
| 35-49 | 1.66 | 1.46 | 1.88 | 0.00 | 1.72 | 1.43 | 2.07 | 0.00 | 1.06 | 0.92 | 1.22 | 0.43 | 1.08 | 0.85 | 1.37 | 0.52 |
| **State classification** |  |  |  |  |  |  |  |  |  |  |  |  |  |  |  |  |
| EAG states | Ref |  |  |  | Ref |  |  |  | Ref |  |  |  | Ref |  |  |  |
| Non EAG states | 1.73 | 0.99 | 3.01 | 0.05 | 1.47 | 0.91 | 2.38 | 0.12 | 1.41 | 0.94 | 2.12 | 0.10 | 1.21 | 0.77 | 1.9 | 0.40 |
| **Religion** |  |  |  |  |  |  |  |  |  |  |  |  |  |  |  |  |
| Hindu | Ref |  |  |  | Ref |  |  |  | Ref |  |  |  | Ref |  |  |  |
| Christian | 0.74 | 0.61 | 0.91 | 0.00 | 0.82 | 0.61 | 1.11 | 0.20 | 0.77 | 0.61 | 0.96 | 0.02 | 0.69 | 0.48 | 1 | 0.05 |
| Muslim | 0.88 | 0.77 | 1 | 0.04 | 1 | 0.87 | 1.15 | 0.97 | 1.07 | 0.93 | 1.24 | 0.34 | 1.09 | 0.91 | 1.3 | 0.36 |
| Other | 1.12 | 0.92 | 1.35 | 0.25 | 1.04 | 0.79 | 1.37 | 0.77 | 0.95 | 0.76 | 1.2 | 0.69 | 1.05 | 0.73 | 1.52 | 0.78 |
| **Parity** |  |  |  |  |  |  |  |  |  |  |  |  |  |  |  |  |
| Two children | Ref |  |  |  | Ref |  |  |  | Ref |  |  |  | Ref |  |  |  |
| More than 2 | 0.73 | 0.67 | 0.79 | 0.00 | 0.75 | 0.66 | 0.85 | 0.00 | 0.83 | 0.75 | 0.91 | 0.00 | 0.76 | 0.64 | 0.89 | 0.00 |
| One child | 2.31 | 2.14 | 2.5 | 0.00 | 1.93 | 1.73 | 2.16 | 0.00 | 1.15 | 1.05 | 1.26 | 0.00 | 1.11 | 0.96 | 1.28 | 0.17 |
| **Caste** |  |  |  |  |  |  |  |  |  |  |  |  |  |  |  |  |
| General category / No caste | Ref |  |  |  | Ref |  |  |  | Ref |  |  |  | Ref |  |  |  |
| Other Backward castes | 0.97 | 0.84 | 1.11 | 0.62 | 0.95 | 0.77 | 1.18 | 0.65 | 0.99 | 0.85 | 1.16 | 0.91 | 1.11 | 0.85 | 1.45 | 0.44 |
| Schedule castes | 1.06 | 0.91 | 1.23 | 0.45 | 0.93 | 0.71 | 1.2 | 0.57 | 0.96 | 0.81 | 1.14 | 0.63 | 1 | 0.73 | 1.39 | 0.98 |
| Schedule tribes | 1.03 | 0.88 | 1.2 | 0.74 | 0.94 | 0.68 | 1.31 | 0.73 | 0.92 | 0.77 | 1.11 | 0.39 | 0.73 | 0.48 | 1.08 | 0.12 |
| **Education** |  |  |  |  |  |  |  |  |  |  |  |  |  |  |  |  |
| No education | Ref |  |  |  | Ref |  |  |  | Ref |  |  |  | Ref |  |  |  |
| Primary | 1.16 | 1.02 | 1.31 | 0.02 | 1.1 | 0.85 | 1.42 | 0.47 | 1.03 | 0.89 | 1.18 | 0.72 | 0.98 | 0.72 | 1.32 | 0.87 |
| Secondary | 1.42 | 1.27 | 1.58 | 0.00 | 1.24 | 1 | 1.53 | 0.05 | 1.22 | 1.08 | 1.38 | 0.00 | 1.14 | 0.89 | 1.47 | 0.31 |
| Higher | 1.6 | 1.19 | 2.15 | 0.00 | 1.55 | 1.03 | 2.31 | 0.03 | 1.04 | 0.74 | 1.45 | 0.82 | 1.06 | 0.63 | 1.77 | 0.83 |
|  |  |  |  |  |  |  |  |  |  |  |  |  |  |  |  |  |
| **Interaction terms** |  |  |  |  |  |  |  |  |  |  |  |  |  |  |  |  |
| **Interaction with wealth** |  |  |  |  |  |  |  |  |  |  |  |  |  |  |  |  |
| Mobile access*Poorest | Ref |  |  |  | Ref |  |  |  | Ref |  |  |  | Ref |  |  |  |
| Mobile access*Poorer | 0.98 | 0.81 | 1.2 | 0.86 | 1.3 | 0.65 | 2.59 | 0.46 | 0.95 | 0.77 | 1.16 | 0.60 | 2.52 | 1.17 | 5.47 | 0.02 |
| Mobile access*Middle | 0.97 | 0.79 | 1.19 | 0.77 | 1.4 | 0.73 | 2.68 | 0.32 | 1.02 | 0.81 | 1.28 | 0.88 | 2.21 | 1.07 | 4.56 | 0.03 |
| Mobile access*Richer | 1.02 | 0.81 | 1.29 | 0.86 | 1.6 | 0.83 | 3.06 | 0.16 | 0.86 | 0.66 | 1.13 | 0.29 | 2.18 | 1.06 | 4.49 | 0.03 |
| Mobile access*Richest | 1.01 | 0.74 | 1.37 | 0.95 | 1.67 | 0.86 | 3.25 | 0.13 | 1.05 | 0.73 | 1.51 | 0.81 | 2.63 | 1.25 | 5.55 | 0.01 |
| **Interaction with education** |  |  |  |  |  |  |  |  |  |  |  |  |  |  |  |  |
| Mobile access*No education | Ref |  |  |  | Ref |  |  |  | Ref |  |  |  | Ref |  |  |  |
| Mobile access*Primary | 0.88 | 0.71 | 1.1 | 0.25 | 0.88 | 0.59 | 1.31 | 0.53 | 1.06 | 0.84 | 1.35 | 0.62 | 1.17 | 0.73 | 1.88 | 0.52 |
| Mobile access*Secondary | 0.85 | 0.71 | 1.01 | 0.07 | 0.95 | 0.69 | 1.32 | 0.77 | 0.96 | 0.79 | 1.16 | 0.66 | 1.15 | 0.78 | 1.69 | 0.48 |
| Mobile access*Higher | 0.8 | 0.57 | 1.13 | 0.20 | 0.87 | 0.54 | 1.41 | 0.58 | 1.32 | 0.89 | 1.96 | 0.16 | 1.65 | 0.9 | 3 | 0.11 |
| **Interaction with caste** |  |  |  |  |  |  |  |  |  |  |  |  |  |  |  |  |
| Mobile access*General category / No caste | Ref |  |  |  | Ref |  |  |  | Ref |  |  |  | Ref |  |  |  |
| Mobile access*Other Backward castes | 1.07 | 0.9 | 1.28 | 0.45 | 0.89 | 0.69 | 1.15 | 0.39 | 0.98 | 0.8 | 1.21 | 0.87 | 0.78 | 0.57 | 1.08 | 0.14 |
| Mobile access*Schedule castes | 1.03 | 0.84 | 1.26 | 0.76 | 0.89 | 0.65 | 1.22 | 0.46 | 0.93 | 0.73 | 1.17 | 0.53 | 0.84 | 0.56 | 1.26 | 0.40 |
| Mobile access*Schedule tribes | 1.14 | 0.93 | 1.4 | 0.19 | 0.83 | 0.57 | 1.22 | 0.34 | 0.95 | 0.75 | 1.2 | 0.66 | 1.13 | 0.7 | 1.8 | 0.62 |

**Table B5. Multilevel models for association of mobile phone access on Delivery and Postnatal care outcomes**

|  | **Skilled birth attendance** | | | | | | | | **Facility Delivery** | | | | | | | | **Postnatal Care** | | | | | | | |
| --- | --- | --- | --- | --- | --- | --- | --- | --- | --- | --- | --- | --- | --- | --- | --- | --- | --- | --- | --- | --- | --- | --- | --- | --- |
|  | **Rural** | | | | **Urban** | | | | **Rural** | | | | **Urban** | | | | **Rural** | | | | **Urban** | | | |
|  | **OR** | **95% CI** | | **p value** | **OR** | **95% CI** | | **p value** | **OR** | **95% CI** | | **p value** | **OR** | **95% CI** | | **p value** | **OR** | **95% CI** | | **p value** | **OR** | **95% CI** | | **p value** |
|  | N=32,548 | | | | N=10,698 | | | | N=32,548 | | | | N=10,698 | | | | N= 23,455 | | | | N= 8,386 | | | |
| **Mobile phone access** |  |  |  |  |  |  |  |  |  |  |  |  |  |  |  |  |  |  |  |  |  |  |  |  |
| No mobile phone access | Ref |  |  |  | Ref |  |  |  | Ref |  |  |  | Ref |  |  |  | Ref |  |  |  | Ref |  |  |  |
| Has access | 1.16 | 0.92 | 1.46 | 0.21 | 1.78 | 0.88 | 3.58 | 0.11 | 1.13 | 0.9 | 1.42 | 0.29 | 1.59 | 0.81 | 3.11 | 0.18 | 0.99 | 0.78 | 1.25 | 0.92 | 2.07 | 1.04 | 4.11 | 0.04 |
| **Multimedia** |  |  |  |  |  |  |  |  |  |  |  |  |  |  |  |  |  |  |  |  |  |  |  |  |
| No exposure | Ref |  |  |  | Ref |  |  |  | Ref |  |  |  | Ref |  |  |  | Ref |  |  |  | Ref |  |  |  |
| Some exposure | 1.18 | 1.08 | 1.29 | 0.00 | 1.25 | 1.01 | 1.55 | 0.04 | 1.18 | 1.08 | 1.29 | 0.00 | 1.24 | 1.02 | 1.51 | 0.03 | 1.44 | 1.32 | 1.57 | 0.00 | 1.39 | 1.14 | 1.69 | 0.00 |
| **Wealth index** |  |  |  |  |  |  |  |  |  |  |  |  |  |  |  |  |  |  |  |  |  |  |  |  |
| Poorest | Ref |  |  |  | Ref |  |  |  | Ref |  |  |  | Ref |  |  |  | Ref |  |  |  | Ref |  |  |  |
| Poorer | 1.47 | 1.32 | 1.64 | 0.00 | 1.59 | 1.11 | 2.29 | 0.01 | 1.52 | 1.36 | 1.69 | 0.00 | 1.35 | 0.95 | 1.92 | 0.09 | 1.38 | 1.19 | 1.6 | 0.00 | 1.61 | 1.1 | 2.37 | 0.01 |
| Middle | 2.24 | 1.92 | 2.6 | 0.00 | 2.39 | 1.63 | 3.52 | 0.00 | 2.15 | 1.86 | 2.49 | 0.00 | 2.04 | 1.41 | 2.95 | 0.00 | 1.22 | 1.09 | 1.37 | 0.00 | 1.29 | 0.88 | 1.88 | 0.20 |
| Richer | 2.67 | 2.13 | 3.36 | 0.00 | 3.38 | 2.24 | 5.1 | 0.00 | 2.4 | 1.93 | 2.98 | 0.00 | 2.58 | 1.75 | 3.82 | 0.00 | 1.48 | 1.21 | 1.82 | 0.00 | 1.60 | 1.08 | 2.37 | 0.02 |
| Richest | 2.64 | 1.83 | 3.81 | 0.00 | 5.4 | 3.27 | 8.9 | 0.00 | 3.39 | 2.35 | 4.9 | 0.00 | 3.49 | 2.2 | 5.55 | 0.00 | 1.91 | 1.39 | 2.63 | 0.00 | 2.00 | 1.29 | 3.1 | 0.00 |
| **Age** |  |  |  |  |  |  |  |  |  |  |  |  |  |  |  |  |  |  |  |  |  |  |  |  |
| 15-24 | Ref |  |  |  | Ref |  |  |  | Ref |  |  |  | Ref |  |  |  | Ref |  |  |  | Ref |  |  |  |
| 25-34 | 1.08 | 0.99 | 1.18 | 0.09 | 1.01 | 0.82 | 1.24 | 0.91 | 1.04 | 0.95 | 1.13 | 0.39 | 1.26 | 1.04 | 1.53 | 0.02 | 1.17 | 1.07 | 1.27 | 0.00 | 1.11 | 0.95 | 1.31 | 0.19 |
| 35-49 | 0.91 | 0.8 | 1.04 | 0.15 | 1.14 | 0.83 | 1.55 | 0.42 | 0.86 | 0.76 | 0.98 | 0.03 | 1.38 | 1.04 | 1.84 | 0.03 | 1.02 | 0.9 | 1.17 | 0.74 | 1.23 | 0.96 | 1.57 | 0.10 |
| **State classification** |  |  |  |  |  |  |  |  |  |  |  |  |  |  |  |  |  |  |  |  |  |  |  |  |
| EAG states | Ref |  |  |  | Ref |  |  |  | Ref |  |  |  | Ref |  |  |  | Ref |  |  |  | Ref |  |  |  |
| Non EAG states | 1.47 | 0.6 | 3.61 | 0.40 | 2.07 | 1.13 | 3.77 | 0.02 | 1.51 | 0.61 | 3.72 | 0.37 | 2.11 | 1 | 4.47 | 0.05 | 1.51 | 1.36 | 1.68 | 0.00 | 1.66 | 1.40 | 1.95 | 0.00 |
| **Religion** |  |  |  |  |  |  |  |  |  |  |  |  |  |  |  |  |  |  |  |  |  |  |  |  |
| Hindu | Ref |  |  |  | Ref |  |  |  | Ref |  |  |  | Ref |  |  |  | Ref |  |  |  | Ref |  |  |  |
| Christian | 0.77 | 0.59 | 0.99 | 0.04 | 0.68 | 0.37 | 1.25 | 0.21 | 0.72 | 0.56 | 0.92 | 0.01 | 0.62 | 0.35 | 1.1 | 0.10 | 0.45 | 0.38 | 0.54 | 0.00 | 0.65 | 0.46 | 0.92 | 0.01 |
| Muslim | 0.61 | 0.53 | 0.7 | 0.00 | 0.79 | 0.63 | 1.01 | 0.06 | 0.6 | 0.53 | 0.69 | 0.00 | 0.76 | 0.61 | 0.95 | 0.01 | 0.79 | 0.69 | 0.91 | 0.00 | 1.06 | 0.88 | 1.28 | 0.53 |
| Other | 0.97 | 0.75 | 1.26 | 0.83 | 1.46 | 0.77 | 2.77 | 0.25 | 0.96 | 0.75 | 1.24 | 0.77 | 1.24 | 0.71 | 2.14 | 0.45 | 0.94 | 0.76 | 1.16 | 0.58 | 1.2 | 0.80 | 1.79 | 0.38 |
| **Parity** |  |  |  |  |  |  |  |  |  |  |  |  |  |  |  |  |  |  |  |  |  |  |  |  |
| Two children | Ref |  |  |  | Ref |  |  |  | Ref |  |  |  | Ref |  |  |  | Ref |  |  |  | Ref |  |  |  |
| more than 2 | 0.6 | 0.55 | 0.65 | 0.00 | 0.64 | 0.52 | 0.78 | 0.00 | 0.57 | 0.53 | 0.62 | 0.00 | 0.59 | 0.49 | 0.71 | 0.00 | 0.76 | 0.69 | 0.83 | 0.00 | 0.73 | 0.62 | 0.86 | 0.00 |
| one child | 1.86 | 1.67 | 2.08 | 0.00 | 1.39 | 1.1 | 1.77 | 0.01 | 2.05 | 1.84 | 2.28 | 0.00 | 1.84 | 1.46 | 2.31 | 0.00 | 1.28 | 1.16 | 1.4 | 0.00 | 1.12 | 0.96 | 1.31 | 0.16 |
| **Caste** |  |  |  |  |  |  |  |  |  |  |  |  |  |  |  |  |  |  |  |  |  |  |  |  |
| General category / No caste | Ref |  |  |  | Ref |  |  |  | Ref |  |  |  | Ref |  |  |  | Ref |  |  |  | Ref |  |  |  |
| Other Backward castes | 0.98 | 0.83 | 1.14 | 0.77 | 0.93 | 0.67 | 1.3 | 0.67 | 0.95 | 0.81 | 1.11 | 0.52 | 0.84 | 0.61 | 1.14 | 0.26 | 1.07 | 0.91 | 1.25 | 0.41 | 0.99 | 0.75 | 1.3 | 0.93 |
| Schedule castes | 0.83 | 0.7 | 0.99 | 0.04 | 0.71 | 0.48 | 1.04 | 0.08 | 0.86 | 0.73 | 1.02 | 0.08 | 0.87 | 0.6 | 1.25 | 0.45 | 1.02 | 0.86 | 1.21 | 0.80 | 1.26 | 0.9 | 1.77 | 0.18 |
| Schedule tribes | 0.53 | 0.44 | 0.63 | 0.00 | 0.69 | 0.42 | 1.13 | 0.14 | 0.45 | 0.38 | 0.54 | 0.00 | 0.48 | 0.31 | 0.76 | 0.00 | 0.92 | 0.77 | 1.09 | 0.34 | 1.31 | 0.86 | 2.00 | 0.21 |
| **Education** |  |  |  |  |  |  |  |  |  |  |  |  |  |  |  |  |  |  |  |  |  |  |  |  |
| No education | Ref |  |  |  | Ref |  |  |  | Ref |  |  |  | Ref |  |  |  | Ref |  |  |  | Ref |  |  |  |
| Primary | 1.24 | 1.1 | 1.4 | 0.00 | 1.29 | 0.95 | 1.74 | 0.10 | 1.15 | 1.02 | 1.3 | 0.02 | 1.28 | 0.96 | 1.7 | 0.09 | 1.31 | 1.17 | 1.47 | 0.00 | 1.48 | 1.16 | 1.91 | 0.00 |
| Secondary | 1.54 | 1.38 | 1.73 | 0.00 | 2.01 | 1.53 | 2.65 | 0.00 | 1.53 | 1.36 | 1.71 | 0.00 | 1.99 | 1.54 | 2.58 | 0.00 | 1.63 | 1.13 | 2.34 | 0.01 | 1.76 | 1.02 | 3.03 | 0.04 |
| Higher | 2.62 | 1.65 | 4.18 | 0.00 | 1.91 | 0.93 | 3.95 | 0.08 | 2.42 | 1.55 | 3.77 | 0.00 | 3.05 | 1.45 | 6.42 | 0.00 | 1.12 | 0.99 | 1.28 | 0.08 | 1.25 | 0.93 | 1.68 | 0.14 |
|  |  |  |  |  |  |  |  |  |  |  |  |  |  |  |  |  |  |  |  |  |  |  |  |  |
| **Interaction terms** |  |  |  |  |  |  |  |  |  |  |  |  |  |  |  |  |  |  |  |  |  |  |  |  |
| **Interaction with wealth** |  |  |  |  |  |  |  |  |  |  |  |  |  |  |  |  |  |  |  |  |  |  |  |  |
| Mobile access*Poorest | Ref |  |  |  | Ref |  |  |  | Ref |  |  |  | Ref |  |  |  | Ref |  |  |  | Ref |  |  |  |
| Mobile access*Poorer | 0.86 | 0.71 | 1.03 | 0.09 | 0.66 | 0.32 | 1.32 | 0.24 | 0.89 | 0.75 | 1.07 | 0.23 | 0.7 | 0.36 | 1.38 | 0.30 | 1.12 | 0.9 | 1.4 | 0.30 | 0.55 | 0.28 | 1.09 | 0.09 |
| Mobile access*Middle | 0.89 | 0.71 | 1.11 | 0.31 | 0.54 | 0.28 | 1.06 | 0.07 | 0.89 | 0.72 | 1.11 | 0.30 | 0.53 | 0.28 | 1 | 0.05 | 0.82 | 0.67 | 0.99 | 0.04 | 0.77 | 0.37 | 1.58 | 0.47 |
| Mobile access*Richer | 1.11 | 0.83 | 1.5 | 0.48 | 0.92 | 0.46 | 1.81 | 0.80 | 1.25 | 0.94 | 1.67 | 0.12 | 0.83 | 0.43 | 1.59 | 0.57 | 1.37 | 1.04 | 1.79 | 0.02 | 0.77 | 0.39 | 1.51 | 0.45 |
| Mobile access*Richest | 1.44 | 0.92 | 2.25 | 0.11 | 0.71 | 0.34 | 1.5 | 0.37 | 1.45 | 0.92 | 2.26 | 0.11 | 0.86 | 0.42 | 1.73 | 0.67 | 1.42 | 0.96 | 2.09 | 0.08 | 0.72 | 0.36 | 1.45 | 0.36 |
| **Interaction with education** |  |  |  |  |  |  |  |  |  |  |  |  |  |  |  |  |  |  |  |  |  |  |  |  |
| Mobile access*No education | Ref |  |  |  | Ref |  |  |  | Ref |  |  |  | Ref |  |  |  | Ref |  |  |  | Ref |  |  |  |
| Mobile access*Primary | 1.01 | 0.82 | 1.25 | 0.92 | 1.27 | 0.77 | 2.11 | 0.35 | 1.03 | 0.83 | 1.27 | 0.80 | 1.06 | 0.67 | 1.69 | 0.80 | 1.1 | 0.92 | 1.33 | 0.30 | 1.09 | 0.74 | 1.6 | 0.66 |
| Mobile access*Secondary | 1.07 | 0.89 | 1.28 | 0.47 | 1.03 | 0.68 | 1.57 | 0.89 | 1.1 | 0.92 | 1.31 | 0.30 | 1.13 | 0.76 | 1.66 | 0.55 | 1.06 | 0.69 | 1.61 | 0.80 | 1.25 | 0.66 | 2.36 | 0.49 |
| Mobile access*Higher | 1.22 | 0.71 | 2.1 | 0.47 | 2.41 | 1.02 | 5.71 | 0.05 | 1.41 | 0.84 | 2.37 | 0.20 | 1.8 | 0.76 | 4.29 | 0.18 | 1.18 | 0.94 | 1.48 | 0.16 | 0.94 | 0.58 | 1.51 | 0.79 |
| **Interaction with caste** |  |  |  |  |  |  |  |  |  |  |  |  |  |  |  |  |  |  |  |  |  |  |  |  |
| Mobile access*General category / No caste | Ref |  |  |  | Ref |  |  |  | Ref |  |  |  | Ref |  |  |  | Ref |  |  |  | Ref |  |  |  |
| Mobile access*Other Backward castes | 0.97 | 0.78 | 1.21 | 0.81 | 0.95 | 0.61 | 1.49 | 0.84 | 1 | 0.81 | 1.24 | 0.98 | 1.09 | 0.71 | 1.65 | 0.70 | 1.00 | 0.81 | 1.23 | 0.97 | 0.81 | 0.58 | 1.14 | 0.24 |
| Mobile access*Schedule castes | 0.9 | 0.7 | 1.14 | 0.38 | 1.08 | 0.63 | 1.84 | 0.78 | 0.87 | 0.69 | 1.11 | 0.26 | 0.8 | 0.48 | 1.32 | 0.38 | 1.02 | 0.8 | 1.29 | 0.89 | 0.67 | 0.44 | 1.02 | 0.06 |
| Mobile access*Schedule tribes | 1.31 | 1.04 | 1.67 | 0.02 | 1.1 | 0.59 | 2.03 | 0.76 | 1.37 | 1.08 | 1.73 | 0.01 | 1.07 | 0.61 | 1.88 | 0.81 | 0.93 | 0.73 | 1.17 | 0.53 | 0.61 | 0.37 | 1.01 | 0.05 |

**Table B6. Multilevel models for association of mobile phone access on family planning outcomes**

|  | **Modern Contraceptive use** | | | | | | | | **Unmet need** | | | | | | | |
| --- | --- | --- | --- | --- | --- | --- | --- | --- | --- | --- | --- | --- | --- | --- | --- | --- |
|  | **Rural** | | | | **Urban** | | | | **Rural** | | | | **Urban** | | | |
|  | **OR** | **95% CI** | | **p value** | **OR** | **95% CI** | | **p value** | **OR** | **95% CI** | | **p value** | **OR** | **95% CI** | | **p value** |
|  | N=32548 | | | | N=10698 | | | | N=32223 | | | | N=10585 | | | |
| **Mobile phone access** |  |  |  |  |  |  |  |  |  |  |  |  |  |  |  |  |
| No mobile phone access | Ref |  |  |  | Ref |  |  |  | Ref |  |  |  | Ref |  |  |  |
| Has access | 1.13 | 0.91 | 1.4 | 0.26 | 1.84 | 1.02 | 3.33 | 0.00 | 1.14 | 0.93 | 1.39 | 0.21 | 0.92 | 0.5 | 1.67 | 0.77 |
| **Multimedia** |  |  |  |  |  |  |  |  |  |  |  |  |  |  |  |  |
| No exposure | Ref |  |  |  | Ref |  |  |  | Ref |  |  |  | Ref |  |  |  |
| Some exposure | 1.33 | 1.23 | 1.44 | 0.00 | 1.43 | 1.2 | 1.7 | 0.00 | 0.93 | 0.87 | 1.01 | 0.09 | 0.91 | 0.77 | 1.08 | 0.30 |
| **Wealth index** |  |  |  |  |  |  |  |  |  |  |  |  |  |  |  |  |
| Poorest | Ref |  |  |  | Ref |  |  |  | Ref |  |  |  | Ref |  |  |  |
| Poorer | 1.22 | 1.1 | 1.36 | 0.00 | 1.15 | 0.83 | 1.61 | 0.40 | 0.99 | 0.9 | 1.1 | 0.91 | 1.59 | 1.12 | 2.25 | 0.01 |
| Middle | 1.34 | 1.18 | 1.53 | 0.00 | 1.03 | 0.74 | 1.45 | 0.90 | 1.08 | 0.95 | 1.23 | 0.24 | 2.03 | 1.43 | 2.89 | 0.00 |
| Richer | 1.5 | 1.26 | 1.79 | 0.00 | 1.25 | 0.88 | 1.77 | 0.20 | 0.9 | 0.75 | 1.08 | 0.27 | 1.53 | 1.06 | 2.21 | 0.02 |
| Richest | 1.25 | 0.96 | 1.63 | 0.09 | 1.25 | 0.86 | 1.83 | 0.20 | 1.44 | 1.1 | 1.87 | 0.01 | 1.73 | 1.16 | 2.58 | 0.01 |
| **Age** |  |  |  |  |  |  |  |  |  |  |  |  |  |  |  |  |
| 15-24 | Ref |  |  |  | Ref |  |  |  | Ref |  |  |  | Ref |  |  |  |
| 25-34 | 1.42 | 1.32 | 1.53 | 0.00 | 1.19 | 1.05 | 1.35 | 0.00 | 0.95 | 0.88 | 1.02 | 0.14 | 0.82 | 0.72 | 0.93 | 0.00 |
| 35-49 | 1.27 | 1.12 | 1.43 | 0.00 | 1.1 | 0.91 | 1.34 | 0.30 | 1.25 | 1.12 | 1.4 | 0.00 | 1.19 | 0.97 | 1.45 | 0.09 |
| **State classification** |  |  |  |  |  |  |  |  |  |  |  |  |  |  |  |  |
| EAG states | Ref |  |  |  | Ref |  |  |  | Ref |  |  |  | Ref |  |  |  |
| Non EAG states | 1.48 | 0.77 | 2.84 | 0.24 | 1.22 | 0.74 | 2.02 | 0.50 | 0.77 | 0.49 | 1.2 | 0.24 | 0.99 | 0.59 | 1.66 | 0.97 |
| **Religion** |  |  |  |  |  |  |  |  |  |  |  |  |  |  |  |  |
| Hindu | Ref |  |  |  | Ref |  |  |  | Ref |  |  |  | Ref |  |  |  |
| Christian | 0.66 | 0.52 | 0.83 | 0.00 | 0.58 | 0.41 | 0.82 | 0.00 | 1.27 | 1.03 | 1.56 | 0.02 | 1.47 | 1.06 | 2.04 | 0.02 |
| Muslim | 0.62 | 0.54 | 0.7 | 0.00 | 0.76 | 0.65 | 0.89 | 0.00 | 1.23 | 1.09 | 1.38 | 0.00 | 0.9 | 0.77 | 1.06 | 0.21 |
| Other | 0.86 | 0.69 | 1.07 | 0.18 | 0.66 | 0.48 | 0.9 | 0.00 | 1.19 | 0.97 | 1.46 | 0.10 | 1.35 | 0.97 | 1.86 | 0.07 |
| **Parity** |  |  |  |  |  |  |  |  |  |  |  |  |  |  |  |  |
| Two children | Ref |  |  |  | Ref |  |  |  | Ref |  |  |  | Ref |  |  |  |
| More than 2 | 1.49 | 1.38 | 1.61 | 0.00 | 1.36 | 1.2 | 1.55 | 0.00 | 1.07 | 0.99 | 1.15 | 0.10 | 0.94 | 0.82 | 1.07 | 0.35 |
| One child | 0.27 | 0.25 | 0.3 | 0.00 | 0.31 | 0.27 | 0.35 | 0.00 | 1.11 | 1.03 | 1.21 | 0.01 | 1.15 | 1.01 | 1.31 | 0.03 |
| **Caste** |  |  |  |  |  |  |  |  |  |  |  |  |  |  |  |  |
| General category / No caste | Ref |  |  |  | Ref |  |  |  | Ref |  |  |  | Ref |  |  |  |
| Other Backward castes | 1.09 | 0.94 | 1.25 | 0.25 | 1.09 | 0.87 | 1.38 | 0.50 | 0.88 | 0.77 | 1.01 | 0.07 | 1.01 | 0.79 | 1.28 | 0.94 |
| Schedule castes | 1 | 0.86 | 1.16 | 0.99 | 1.31 | 1 | 1.73 | 0.10 | 0.95 | 0.82 | 1.1 | 0.51 | 0.7 | 0.52 | 0.94 | 0.02 |
| Schedule tribes | 0.82 | 0.69 | 0.96 | 0.02 | 0.89 | 0.62 | 1.28 | 0.50 | 0.92 | 0.78 | 1.08 | 0.28 | 0.78 | 0.54 | 1.14 | 0.20 |
| **Education** |  |  |  |  |  |  |  |  |  |  |  |  |  |  |  |  |
| No education | Ref |  |  |  | Ref |  |  |  | Ref |  |  |  | Ref |  |  |  |
| Primary | 1.22 | 1.09 | 1.37 | 0.00 | 1.44 | 1.13 | 1.84 | 0.00 | 0.92 | 0.82 | 1.03 | 0.15 | 0.6 | 0.46 | 0.78 | 0.00 |
| Secondary | 1.18 | 1.07 | 1.31 | 0.00 | 1.32 | 1.07 | 1.63 | 0.00 | 1.06 | 0.96 | 1.18 | 0.24 | 0.78 | 0.62 | 0.96 | 0.02 |
| Higher | 1.35 | 0.99 | 1.85 | 0.06 | 1.34 | 0.86 | 2.08 | 0.20 | 1 | 0.74 | 1.36 | 0.99 | 0.94 | 0.6 | 1.47 | 0.79 |
|  |  |  |  |  |  |  |  |  |  |  |  |  |  |  |  |  |
| **Interaction terms** |  |  |  |  |  |  |  |  |  |  |  |  |  |  |  |  |
| **Interaction with wealth** |  |  |  |  |  |  |  |  |  |  |  |  |  |  |  |  |
| Mobile access*Poorest | Ref |  |  |  | Ref |  |  |  | Ref |  |  |  | Ref |  |  |  |
| Mobile access*Poorer | 0.91 | 0.76 | 1.09 | 0.30 | 0.57 | 0.3 | 1.06 | 0.10 | 0.96 | 0.82 | 1.14 | 0.67 | 0.73 | 0.39 | 1.37 | 0.33 |
| Mobile access*Middle | 0.95 | 0.78 | 1.16 | 0.62 | 0.67 | 0.37 | 1.21 | 0.20 | 0.86 | 0.72 | 1.04 | 0.12 | 0.68 | 0.38 | 1.23 | 0.21 |
| Mobile access*Richer | 0.93 | 0.73 | 1.17 | 0.53 | 0.63 | 0.35 | 1.12 | 0.10 | 0.96 | 0.76 | 1.21 | 0.73 | 0.83 | 0.46 | 1.5 | 0.54 |
| Mobile access*Richest | 1.12 | 0.82 | 1.53 | 0.49 | 0.75 | 0.41 | 1.36 | 0.30 | 0.67 | 0.49 | 0.92 | 0.01 | 0.66 | 0.36 | 1.22 | 0.19 |
| **Interaction with education** |  |  |  |  |  |  |  |  |  |  |  |  |  |  |  |  |
| Mobile access*No education | Ref |  |  |  | Ref |  |  |  | Ref |  |  |  | Ref |  |  |  |
| Mobile access*Primary | 0.93 | 0.76 | 1.14 | 0.48 | 0.59 | 0.39 | 0.87 | 0.00 | 1.02 | 0.84 | 1.25 | 0.81 | 2.35 | 1.54 | 3.57 | 0.00 |
| Mobile access*Secondary | 1 | 0.84 | 1.19 | 1.00 | 0.98 | 0.71 | 1.35 | 0.90 | 1.09 | 0.93 | 1.27 | 0.31 | 1.65 | 1.18 | 2.31 | 0.00 |
| Mobile access*Higher | 0.84 | 0.59 | 1.21 | 0.35 | 1.12 | 0.67 | 1.88 | 0.70 | 1.43 | 1 | 2.02 | 0.05 | 1.31 | 0.78 | 2.23 | 0.31 |
| **Interaction with caste** |  |  |  |  |  |  |  |  |  |  |  |  |  |  |  |  |
| Mobile access*General category / No caste | Ref |  |  |  | Ref |  |  |  | Ref |  |  |  | Ref |  |  |  |
| Mobile access*Other Backward castes | 0.86 | 0.72 | 1.03 | 0.09 | 0.95 | 0.72 | 1.24 | 0.70 | 1.12 | 0.94 | 1.34 | 0.20 | 0.9 | 0.68 | 1.19 | 0.44 |
| Mobile access*Schedule castes | 0.9 | 0.73 | 1.1 | 0.28 | 0.64 | 0.46 | 0.9 | 0.00 | 1.05 | 0.86 | 1.28 | 0.65 | 1.19 | 0.83 | 1.71 | 0.34 |
| Mobile access*Schedule tribes | 1.05 | 0.85 | 1.3 | 0.62 | 0.94 | 0.62 | 1.43 | 0.80 | 0.92 | 0.75 | 1.13 | 0.44 | 1.26 | 0.83 | 1.91 | 0.28 |

**Table 7. Multilevel models for association of mobile phone access on child outcomes**

|  | **Full immunization** | | | | | | | | **Children under age 6 months exclusively breastfed** | | | | | | | |
| --- | --- | --- | --- | --- | --- | --- | --- | --- | --- | --- | --- | --- | --- | --- | --- | --- |
|  | **Rural** | | | | **Urban** | | | | **Rural** | | | | **Urban** | | | |
|  | **OR** | **95% CI** | | **p value** | **OR** | **95% CI** | | **p value** | **OR** | **95% CI** | | **p value** | **OR** | **95% CI** | | **p value** |
|  | N=30800 | | | | N=10284 | | | | **N=3416** | | | | **N=1090** | | | |
| **Mobile phone access** |  |  |  |  |  |  |  |  |  |  |  |  |  |  |  |  |
| No mobile phone access | Ref |  |  |  | Ref |  |  |  | Ref |  |  |  | Ref |  |  |  |
| Has access | 1.01 | 0.86 | 1.2 | 0.87 | 1.36 | 0.82 | 2.27 | 0.23 | 0.73 | 0.35 | 1.54 | 0.41 | 0.98 | 0.09 | 11.16 | 0.99 |
| **Multimedia** |  |  |  |  |  |  |  |  |  |  |  |  |  |  |  |  |
| No exposure | Ref |  |  |  | Ref |  |  |  | Ref |  |  |  | Ref |  |  |  |
| Some exposure | 1.09 | 1.01 | 1.17 | 0.02 | 1.21 | 1.04 | 1.4 | 0.01 | 0.8 | 0.61 | 1.04 | 0.09 | 0.78 | 0.43 | 1.42 | 0.41 |
| **Wealth index** |  |  |  |  |  |  |  |  |  |  |  |  |  |  |  |  |
| poorest | Ref |  |  |  | Ref |  |  |  | Ref |  |  |  | Ref |  |  |  |
| poorer | 1.22 | 1.11 | 1.33 | 0.00 | 1.09 | 0.81 | 1.46 | 0.57 | 1.03 | 0.73 | 1.45 | 0.87 | 1.00 | 0.31 | 3.27 | 1.00 |
| middle | 1.42 | 1.27 | 1.59 | 0.00 | 1.31 | 0.97 | 1.76 | 0.07 | 1.05 | 0.68 | 1.61 | 0.82 | 0.70 | 0.22 | 2.22 | 0.54 |
| richer | 1.51 | 1.3 | 1.76 | 0.00 | 1.27 | 0.94 | 1.72 | 0.12 | 0.85 | 0.46 | 1.55 | 0.59 | 1.17 | 0.38 | 3.63 | 0.79 |
| richest | 1.42 | 1.13 | 1.8 | 0.00 | 1.38 | 0.99 | 1.9 | 0.05 | 0.66 | 0.26 | 1.67 | 0.38 | 1.03 | 0.31 | 3.42 | 0.96 |
| **Age** |  |  |  |  |  |  |  |  |  |  |  |  |  |  |  |  |
| 15-24 | Ref |  |  |  | Ref |  |  |  | Ref |  |  |  | Ref |  |  |  |
| 25-34 | 1.35 | 1.26 | 1.43 | 0.00 | 1.39 | 1.24 | 1.55 | 0.00 | 0.92 | 0.72 | 1.16 | 0.47 | 1.48 | 0.98 | 2.25 | 0.06 |
| 35-49 | 1.33 | 1.2 | 1.48 | 0.00 | 1.76 | 1.47 | 2.11 | 0.00 | 1.91 | 1.24 | 2.94 | 0.00 | 0.61 | 0.24 | 1.54 | 0.30 |
| **State classification** |  |  |  |  |  |  |  |  |  |  |  |  |  |  |  |  |
| EAG states | Ref |  |  |  | Ref |  |  |  | Ref |  |  |  | Ref |  |  |  |
| non EAG states | 0.93 | 0.61 | 1.41 | 0.73 | 0.95 | 0.67 | 1.35 | 0.77 | 2.41 |  | 3.63 | 0.00 | 1.61 | 1.09 | 2.38 | 0.02 |
| **Religion** |  |  |  |  |  |  |  |  |  |  |  |  |  |  |  |  |
| Hindu | Ref |  |  |  | Ref |  |  |  | Ref |  |  |  | Ref |  |  |  |
| Christian | 0.95 | 0.79 | 1.14 | 0.58 | 0.63 | 0.48 | 0.84 | 0.00 | 0.86 | 0.52 | 1.42 | 0.55 | 3.00 | 1.31 | 6.87 | 0.01 |
| Muslim | 0.72 | 0.65 | 0.8 | 0.00 | 0.84 | 0.73 | 0.96 | 0.01 | 1.15 | 0.81 | 1.63 | 0.45 | 0.81 | 0.49 | 1.34 | 0.42 |
| other religion | 1.27 | 1.06 | 1.53 | 0.01 | 0.97 | 0.74 | 1.29 | 0.85 | 0.89 | 0.50 | 1.56 | 0.67 | 0.13 | 0.02 | 0.99 | 0.05 |
| **Parity** |  |  |  |  |  |  |  |  |  |  |  |  |  |  |  |  |
| Two children | Ref |  |  |  | Ref |  |  |  | Ref |  |  |  | Ref |  |  |  |
| More than 2 | 0.82 | 0.77 | 0.88 | 0.00 | 0.8 | 0.71 | 0.9 | 0.00 | 0.6 | 0.46 | 0.79 | 0.00 | 0.99 | 0.61 | 1.6 | 0.96 |
| One child | 0.89 | 0.82 | 0.95 | 0.00 | 0.92 | 0.82 | 1.03 | 0.13 | 0.39 | 0.30 | 0.50 | 0.00 | 0.63 | 0.41 | 0.97 | 0.03 |
| **Caste** |  |  |  |  |  |  |  |  |  |  |  |  |  |  |  |  |
| General category / No caste | Ref |  |  |  | Ref |  |  |  | Ref |  |  |  | Ref |  |  |  |
| Other Backward castes | 0.97 | 0.86 | 1.1 | 0.64 | 0.9 | 0.73 | 1.11 | 0.32 | 1.02 | 0.62 | 1.68 | 0.93 | 1.31 | 0.57 | 3.03 | 0.53 |
| schedule caste | 0.91 | 0.79 | 1.03 | 0.14 | 0.86 | 0.67 | 1.11 | 0.25 | 1.3 | 0.78 | 2.19 | 0.31 | 1.12 | 0.41 | 3.02 | 0.83 |
| schedule tribe | 0.80 | 0.70 | 0.92 | 0.00 | 0.9 | 0.66 | 1.24 | 0.52 | 1.14 | 0.67 | 1.92 | 0.63 | 0.48 | 0.13 | 1.72 | 0.26 |
| **Education** |  |  |  |  |  |  |  |  |  |  |  |  |  |  |  |  |
| No education | Ref |  |  |  | Ref |  |  |  | Ref |  |  |  | Ref |  |  |  |
| Primary | 1.21 | 1.11 | 1.32 | 0.00 | 1.36 | 1.14 | 1.63 | 0.00 | 1.1 | 0.79 | 1.53 | 0.57 | 3.13 | 1.29 | 7.58 | 0.01 |
| Secondary | 1.31 | 1.21 | 1.41 | 0.00 | 1.27 | 1.09 | 1.48 | 0.00 | 0.9 | 0.67 | 1.21 | 0.48 | 2.56 | 1.17 | 5.58 | 0.02 |
| Higher | 1.22 | 1.06 | 1.39 | 0.00 | 1.26 | 1.04 | 1.54 | 0.02 | 1.31 | 0.81 | 2.1 | 0.27 | 1.96 | 0.79 | 4.83 | 0.14 |
|  |  |  |  |  |  |  |  |  |  |  |  |  |  |  |  |  |
| **Interaction terms** |  |  |  |  |  |  |  |  |  |  |  |  |  |  |  |  |
| **Interaction with wealth** |  |  |  |  |  |  |  |  |  |  |  |  |  |  |  |  |
| Mobile access*Poorest | Ref |  |  |  | Ref |  |  |  | Ref |  |  |  | Ref |  |  |  |
| Mobile access*Poorer | 0.90 | 0.77 | 1.04 | 0.15 | 0.89 | 0.51 | 1.54 | 0.67 | 1.88 | 0.98 | 3.61 | 0.06 | 0.48 | 0.03 | 7.28 | 0.60 |
| Mobile access*Middle | 0.92 | 0.79 | 1.08 | 0.32 | 0.81 | 0.49 | 1.35 | 0.43 | 1.79 | 0.9 | 3.56 | 0.10 | 1.51 | 0.13 | 17.69 | 0.74 |
| Mobile access*Richer | 0.82 | 0.68 | 0.99 | 0.04 | 0.87 | 0.53 | 1.43 | 0.58 | 1.65 | 0.73 | 3.72 | 0.22 | 1.19 | 0.11 | 12.75 | 0.89 |
| Mobile access*Richest | 1.04 | 0.80 | 1.35 | 0.80 | 0.83 | 0.5 | 1.38 | 0.47 | 2.27 | 0.76 | 6.77 | 0.14 | 1.43 | 0.13 | 15.73 | 0.77 |
| Interaction with caste |  |  |  |  |  |  |  |  |  |  |  |  |  |  |  |  |
| Mobile access*General category / No caste | Ref |  |  |  | Ref |  |  |  | Ref |  |  |  | Ref |  |  |  |
| Mobile access*Other Backward castes | 1.11 | 0.95 | 1.29 | 0.21 | 0.97 | 0.76 | 1.23 | 0.79 | 1.00 | 0.54 | 1.88 | 0.99 | 0.63 | 0.23 | 1.69 | 0.36 |
| Mobile access*Schedule castes | 1.12 | 0.94 | 1.34 | 0.22 | 0.92 | 0.68 | 1.25 | 0.61 | 0.63 | 0.31 | 1.28 | 0.20 | 0.34 | 0.09 | 1.29 | 0.11 |
| Mobile access*Schedule tribes | 1.38 | 1.15 | 1.65 | 0.00 | 1.05 | 0.73 | 1.51 | 0.78 | 0.81 | 0.41 | 1.60 | 0.55 | 1.38 | 0.34 | 5.67 | 0.65 |
